# Spatial segregation and bycatch risk as potential drivers of population trends of wandering albatrosses at South Georgia

**DOI:** 10.1111/cobi.70126

**Published:** 2025-08-20

**Authors:** V. Warwick‐Evans, E. J. Pearmain, L. Thorne, R. A. Phillips

**Affiliations:** ^1^ British Antarctic Survey Natural Environment Research Council Cambridge UK; ^2^ School of Marine and Atmospheric Sciences Stony Brook University Stony Brook New York USA

**Keywords:** Fisheries bycatch, fisheries overlap, foraging ecology, pelagic seabirds, population decline, population overlap, aves pelágicas, captura accidental, declinación poblacional, ecología del forrajeo, traslape de pesquerías, traslape poblacional, 渔业兼捕, 渔业重叠, 觅食生态学, 种群下降, 种群重叠, 远洋海鸟

## Abstract

Spatial segregation in at‐sea distribution is frequently observed in seabirds and can have important implications for conservation and management. Globally, many albatross and petrel populations are declining due to bycatch in fisheries. In South Georgia, the decrease in wandering albatrosses (*Diomedea exulans*) differs among breeding sites, which could reflect segregation in foraging areas, leading to differing degrees of overlap with particular fishing fleets and hence unequal bycatch risk. We investigated whether spatial segregation could explain the different rates of population decline of wandering albatrosses at South Georgia. We tracked wandering albatrosses from 2 breeding sites at South Georgia, Prion Island, and Bird Island, located 50 km apart. We investigated potential causes of spatial segregation with species distribution models and by comparing wind conditions among sites. Overlap with fisheries was quantified for each population. Although overall distributions were from the Antarctic to the subtropics, virtually all wandering albatrosses from Bird Island foraged only to the west of the island group, whereas those from Prion Island foraged to the east and west. Preferred habitat characteristics were similar at both colonies, and waters to the east and west provided foraging habitat. Wind conditions when birds departed were also similar at the 2 sites. Because neither habitat specialization nor wind conditions appeared to be factors in the observed spatial segregation among colonies, this segregation likely reflected a combination of past experience, information exchange, and cultural evolution. Breeding birds from both sites overlapped most with Chinese squid jiggers, Argentinian trawlers, and South Korean set (demersal) longliners, but the spatial segregation led to a higher overlap with demersal longline, demersal trawl, and pelagic longline fisheries by wandering albatrosses at Bird Island, which could have resulted in the faster population decline. Ours is one of the first studies to demonstrate how spatial segregation may affect population dynamics, which has important implications for the conservation of this globally threatened species.

## Abstract (es)

Segregación espacial y riesgo de captura accidental como factores potenciales de la tendencia poblacional de albatros errantes en Georgia del Sur

**Resumen:**

La segregación espacial en la distribución se observa con frecuencia en las aves marinas y puede tener importantes implicaciones para la conservación y la gestión. A nivel mundial, muchas poblaciones de albatros y petreles disminuyen debido a las capturas accidentales en la pesca. En Georgia del Sur, la disminución de los albatros errantes (*Diomedea exulans*) difiere entre los sitios de reproducción, lo que reflejaría una segregación en las zonas de alimentación que da lugar a diferentes grados de traslape con determinadas flotas pesqueras y, por lo tanto, a un riesgo desigual de capturas accidentales. Investigamos si la segregación espacial podía explicar las diferentes tasas de disminución de la población de albatros errantes en Georgia del Sur. Realizamos un seguimiento de los albatros errantes de dos lugares de cría en Georgia del Sur, la isla Prion y la isla Bird, situadas a 50 km de distancia entre sí. Investigamos las posibles causas de la segregación espacial con modelos de distribución de especies, comparamos las condiciones del viento entre los distintos lugares y cuantificamos el traslape con las pesquerías para cada población. Aunque la distribución general abarcó desde la Antártida hasta los subtrópicos, prácticamente todos los albatros errantes de la isla Bird se alimentaban solo al oeste del archipiélago, mientras que los de la isla Prion lo hacían al este y al oeste. Las características del hábitat preferido eran similares en ambas colonias y las aguas al este y al oeste proporcionaban un hábitat para la alimentación. Las condiciones del viento cuando las aves partían también eran similares en ambos sitios. Dado que ni la especialización del hábitat ni las condiciones del viento parecían ser factores determinantes en la segregación espacial observada entre las colonias, es probable que esta segregación refleja una combinación de experiencias pasadas, intercambio de información y evolución cultural. Las aves reproductoras de ambos sitios se traslapaban en su mayoría con los pescadores chinos de calamar, los arrastreros argentinos y los palangreros surcoreanos fijos (demersales), pero la segregación espacial provocó un mayor traslape con las pesquerías de palangre demersal, arrastre demersal y palangre pelágico por parte de los albatros errantes de la isla Bird, lo que podría haber provocado un descenso más rápido de la población. Nuestro estudio es uno de los primeros en demostrar cómo la segregación espacial puede afectar a la dinámica de las poblaciones, lo que tiene importantes implicaciones para la conservación de esta especie amenazada a nivel mundial.

## 空间隔离与兼捕风险是南乔治亚岛漂泊信天翁种群数量变化的潜在驱动因素

【摘要】 海鸟种群常存在海上分布的空间隔离, 这对保护与管理具有重要意义。全球范围内, 许多信天翁和海燕的种群都因渔业兼捕而发生数量下降。在南乔治亚岛, 不同繁殖位点的漂泊信天翁(*Diomedea exulans*)种群下降情况存在差异, 这可能反映了觅食区域隔离导致鸟类种群与特定渔船船队不同程度的重叠, 进而引发不均等的兼捕风险。本研究探究了空间隔离是否能解释南乔治亚岛漂泊信天翁种群数量下降速率的差异。我们在南乔治亚岛相距50公里的两个繁殖地(普里昂岛和鸟岛)对漂泊信天翁进行了追踪, 通过物种分布模型及各位点风况的比较, 探讨了空间隔离的潜在成因。研究还量化了各个种群与渔船的重叠程度。我们发现, 尽管漂泊信天翁的整体分布范围从南极一直延伸至亚热带, 但几乎所有鸟岛的个体仅在该群岛以西觅食, 而普里昂岛的个体则在东、西两侧觅食。两处栖息地的栖息地选择特征相似, 且东、西两侧水域均提供觅食栖息地。鸟类离巢时的风况在两个地点也相似。由于栖息地专一性或风况均未被证实能够导致种群间观察到的空间隔离, 因此这种隔离很可能反映了过去经验、信息交流和文化进化的综合影响。来自两个地点的繁殖鸟类与中国的钓鱿鱼船、阿根廷的拖网渔船和韩国的底延绳钓船重叠最多, 但空间隔离导致鸟岛的漂泊信天翁与底延绳钓、底拖网和浮延绳钓渔船的重叠更高, 这可能导致其种群下降更快。本研究是最早揭示空间隔离如何影响种群动态的研究之一, 这对保护漂泊信天翁这一全球濒危物种具有重要意义。**【翻译:胡怡思;审校:聂永刚】**

## INTRODUCTION

Spatial segregation in at‐sea distribution among species, colonies, sexes, age classes, and breeding status is observed frequently in seabirds (Bentley et al., 2023; Bolton et al., 2019; Pettex et al., 2019; Wakefield et al., 2013). This may be a result of specialization in habitat or preferred prey (Granroth‐Wilding & Phillips, 2019; Vilchis et al., 2006), foraging‐site fidelity and memory effects (Grémillet et al., 2004), inequality in travel costs from different colonies (Cairns, 1989), or density‐dependent competition (Ashmole, 1963; Patterson et al., 2022; Wakefield et al., 2013; Wakefield et al., 2017). Spatial segregation may result in variability in the overlap of seabirds with high‐risk areas at sea, such as areas of higher fishing effort, pollution, or environmental extremes (e.g., storms). This variability has implications for the ecology and population dynamics of species and populations. Thus, understanding spatial segregation in seabirds is important so that conservation and management plans focus on relevant spatial and temporal scales.

During the breeding season, seabirds are central‐place foragers (i.e., they return regularly to their colony) (Orians & Pearson, 1979), which limits their spatial distribution and leads to prey depletion in surrounding waters (Ashmole, 1963). Optimal foraging theory is based on the assumption that individuals maximize prey consumption and minimize energetic expenditure (Stephens & Krebs, 1986). Thus, although traveling incurs energetic costs, the expectation is that it increases foraging success as a result of reduced competition with other individuals from the same breeding colony. However, this may also result in increased competition for resources with other species or with conspecifics from other breeding colonies, particularly those nearby (Cairns, 1989). Density‐dependent competition among breeding aggregations may lead to spatial segregation of foraging areas when the potential for competition is high, for example, in large or closely situated colonies (Wakefield et al., 2017). In addition, the overlap among individuals from colonies situated far apart is likely to depend not only on colony size, but also on the relative availability of foraging habitat in surrounding waters (Wakefield et al., 2011).

Spatial segregation between breeding populations has important implications for conservation and management. Seabirds encounter many threats at sea, including bycatch (incidental mortality) in fisheries, which is also a serious threat to many other marine animals (Lewison et al., 2014; Phillips et al., 2016). Interactions between seabirds and fishing vessels and bycatch risk during those interactions are species‐specific (Clay et al., 2019; Louzao et al., 2011). Many seabirds are attracted to vessels by offal and discards, but the benefit of this supplementary food is offset by entanglement, hooking, or collisions with fishing gear. Pelagic seabirds are frequently drowned during gear setting or seriously injured during hauling of longlines, or may strike warp or net monitoring cables on trawlers (Bugoni et al., 2008; Jiménez et al., 2011; Phillips & Wood, 2020; Sullivan et al., 2006). Bycatch can be mitigated effectively by, for example, setting gear at night, using heavier line weighting and bird‐scaring lines in longline fisheries, managing discards, using bird‐scaring lines, and prohibiting third (monitoring) wires in trawl fisheries (Collins et al., 2021; Løkkeborg, 2011; Phillips et al., 2016; Phillips et al., 2024). However, mitigation measures for many fishing fleets are not best practice, and there is insufficient monitoring of compliance. Hence, bycatch rates remain unsustainable for many seabird species (Phillips et al., 2016). Identifying the spatiotemporal overlap of seabirds and fisheries enables the identification of areas of potentially high bycatch risk and should allow mitigation, monitoring, and management to focus on relevant fishing fleets (Clay et al., 2019; Frankish et al., 2021; Reid et al., 2013). Although tracking data from one site are often used to infer at‐sea distributions, and, therefore, bycatch risk, of birds from the island group, if birds from neighboring colonies are segregated spatially or have differing propensity to follow vessels, then estimates of risk from different fleets at particular times of year or for particular ages or sexes may be incorrect.

Albatrosses and petrels are long‐lived seabirds with slow reproductive rates and delayed maturity (Tickell, 1968). Therefore, low adult or juvenile survival rates are often associated with major population declines (Croxall et al., 1990; Pardo et al., 2017). Seabird‐bycatch observer coverage is never complete for wide‐ranging seabirds, but where bycatch rates are known for at least part of the foraging range, these have been implicated in population declines (Dasnon et al., 2022; Genovart et al., 2016; Pardo et al., 2017; Tuck et al., 2003). South Georgia (southwestern Atlantic Ocean) hosts internationally important populations of albatrosses that are decreasing because of interactions with fisheries and climatic change (Carneiro et al. 2022b; Clay et al., 2019; Pardo et al., 2017). Although mitigation measures in the longline fisheries operating in South Georgia waters have reduced seabird bycatch rates to negligible levels (Collins et al., 2021), the albatrosses have huge foraging ranges and are killed in fisheries elsewhere (Clay et al., 2019; Frankish et al., 2021; Jiménez et al., 2014).

The wandering albatross (*Diomedea exulans*) is a wide‐ranging pelagic seabird that forages opportunistically around fishing vessels, and bycatch has resulted in a catastrophic long‐term population decline in South Georgia (Pardo et al., 2017; Poncet et al., 2017). As a result, the wandering albatrosses of South Georgia are listed as 1 of just 9 global high priority populations for conservation under the Agreement on the Conservation of Albatrosses and Petrels (ACAP, 2011). Between 1999 and 2018, the number of breeding pairs of wandering albatrosses at Prion Island and nearby Albatross Island declined by 1.4% per annum (24.1% overall) from 220 to 167 pairs, whereas over the same period, the number at Bird Island declined at twice that rate (3.0% per annum, 44.1% overall) from 1182 to 661 pairs (∼ 60% of the South Georgia population) (Rackete et al., 2021). These varying rates of decline could reflect spatial segregation in foraging areas leading to differing degrees of overlap with fisheries and hence unequal bycatch risk, but, to date, the only tracking has been at Bird Island, which held 60% of the South Georgia population in the 2014−2015 breeding season (Poncet et al., 2017).

We aimed to track breeding wandering albatrosses at Bird Island and Prion Island in the Bay of Isles to determine the degree of spatial segregation and whether relative overlap with particular fisheries might explain the contrasting population trends. In the 2014−2015 breeding season, the colonies in the Bay of Isles contained 16% of the South Georgia population. These colonies are decreasing more slowly than Bird Island colonies (Poncet et al., 2017; Rackete et al., 2021). We also examined possible differences in habitat preferences and compared the strength and direction of wind at the time of departure from Bird Island and Prion Island to establish whether this factor affects the direction of foraging trips. Finally, we calculated the proportion of time wandering albatrosses from each colony spent in fishing areas to quantify colony‐specific bycatch risk. We considered our results in the context of conservation and management initiatives for albatrosses and other pelagic seabirds in general, which are usually based on the assumption that foraging distributions of birds tracked from single colonies represent those of the entire island group.

## METHODS

### Study sites and fieldwork

All fieldwork was approved by the British Antarctic Survey Animal Welfare and Ethical Review Body and carried out under permit from the Government of South Georgia and the South Sandwich Islands.

**FIGURE 1:**
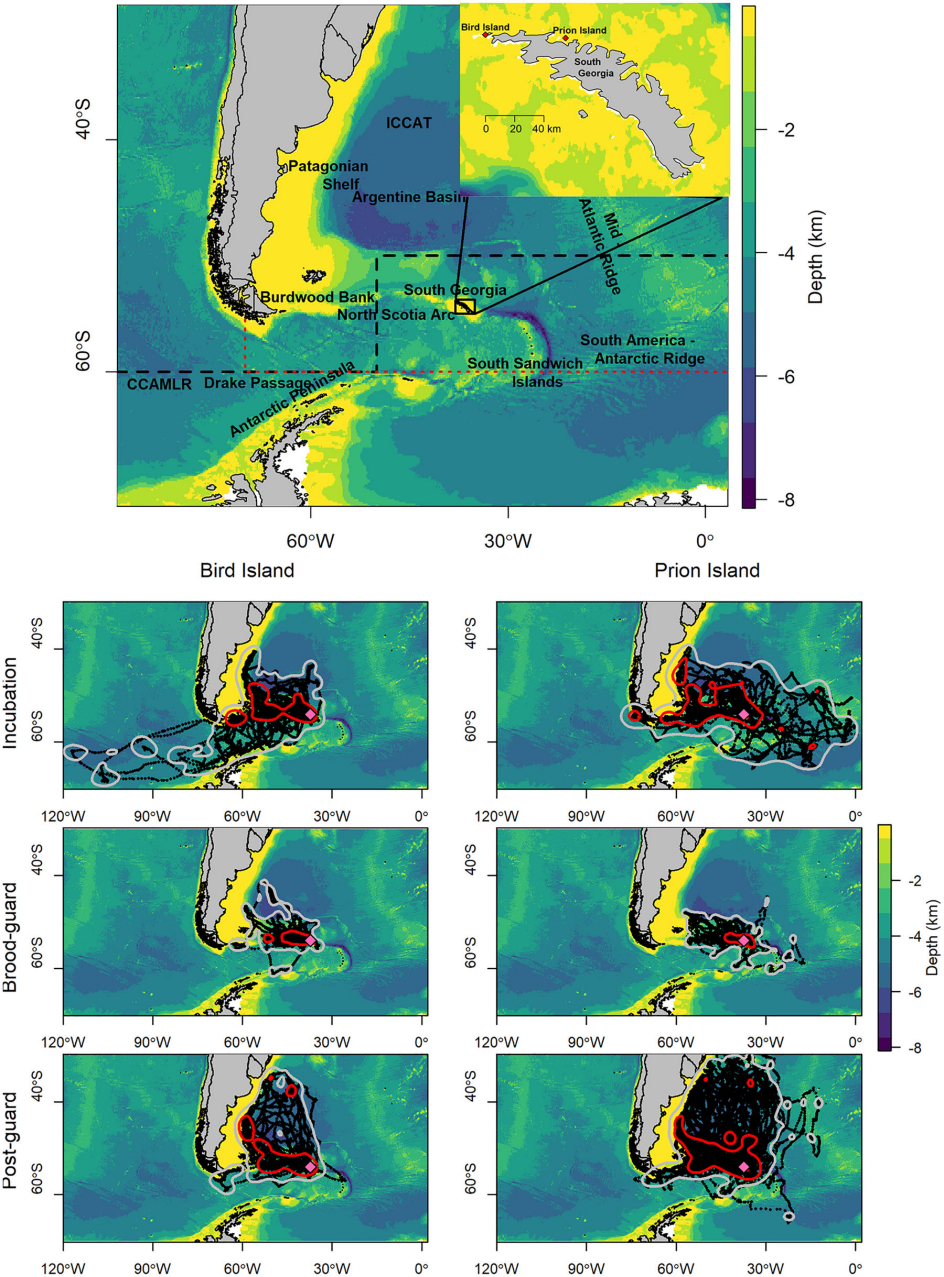
Location of the 2 study colonies of wandering albatrosses in a region of high primary productivity in the southwestern Atlantic Ocean (black dashed line, jurisdiction of Commission for the Conservation of Antarctic Marine Living Resources; red dotted line, jurisdiction of International Commission for the Conservation of Atlantic Tunas) and foraging trips of individuals tracked from Bird Island and Prion Island, South Georgia, during the 2021−2022 breeding season (red polygons, core foraging area [i.e., 50% use distributions]; gray polygons, home range area [95% use distributions]; pink diamond, South Georgia). Tracks include complete and incomplete trips.

We tracked wandering albatrosses from Bird Island (54°00′ S, 38°03′ W) and Prion Island (54°02′ S, 37°25′ W), South Georgia, January−August 2022 (Figure 1). Incubation in wandering albatrosses is from December to March and is followed by the brood‐guard period, when the young chick is brooded alternately by each parent for around 30 days. Following this period is the postguard chick‐rearing period, when the chick can be left unguarded and each parent returns every few days to deliver a meal until the chick fledges in November or December. Hereafter, we refer to our study period as the 2022 breeding season. These islands are <50 km apart, which is a fraction of the maximum foraging range of >2500 km during incubation (Froy et al., 2015; Xavier et al., 2004).

Breeding adults were restrained for <10 min on the nest, and a tracking device was taped to the mantle feathers with Tesa tape (Tesa SE). On Bird Island, GPS loggers (Catlog, 24 g, <0.3% of body mass) set to record locations every 5 min were deployed during incubation and brood‐guard and postguard chick rearing (hereafter postguard) for single trips (with a few exceptions) and recovered when the adult returned to the colony. Access to Prion Island is very limited, and platform terminal transmitters (PTTs) (TAV2664, Telonics) (70 g, < 1% of body mass) were deployed during incubation and left attached for the rest of the season. The PTTs were transmitted every 60 s. Approximately 35 (SD 9) locations per day were provided by the ARGOS satellite system until the PTT batteries failed after 5−8 months. At Prion Island, all 10 PTTs deployed transmitted data. At Bird Island, 30 devices were deployed during each breeding stage, not all of which returned data (Table 1 & Figure 1). Lack of data was attributed to device failures because no birds deserted or nests failed during the deployment periods.

**TABLE 1:** Summary statistics of completed foraging trips of wandering albatrosses tracked from Prion Island and Bird Island, South Georgia, during the 2021−2022 breeding season.

Stage	Colony	Number of birds	Number of trips	Trip duration in hours (SD)	Total distance in kilometers traveled (SD)	Mean foraging range in kilometers (SD)	Bearing to farthest point (°)
Incubation	Bird Island	17	17	310 (77)	6752 (2388)	1947 (964)	260 (31)
	Prion Island	10	25	309 (153)	6911 (4855)	1550 (460)	288 (73)
Brood guard	Bird Island	26	26	78 (26)	2061 (1023)	747 (399)	285 (34)
	Prion Island	10	44	73 (34)	1729 (1321)	538 (433)	297 (100)
Postguard	Bird Island	29	34	191 (134)	4507 (3487)	1282 (821)	295 (44)
	Prion Island	10	118	149 (137)	3851 (3691)	1010 (895)	340 (77)

### Data processing

All data processing and analyses were undertaken with R 4.3.1 (R Core Team, 2021). Raw location data were filtered to remove unrealistic fixes requiring travel speeds of >80 km/h (Froy et al., 2015). The R package crawl was used to estimate location error from the PTTs, and to predict the most likely track with correlated random walks (Johnson, 2013). We subsampled GPS data to regular hourly intervals to broadly match the interval between PTT locations and enable comparisons between colonies. The R package Track2kba (Beal et al. 2021) was used to split the data from Prion Island into individual trips and to calculate summary statistics (trip duration, total distance traveled, foraging range, bearing to farthest point from the colony) for complete trips (i.e., trips that ended in return to the colony) for birds from both colonies. Inclusion of incomplete trips would bias trip summary statistics, so 3 trips from Prion Island and 14 trips from Bird Island were excluded from the between‐site comparison of trip summary statistics. We assessed whether trip duration, foraging range, and total distance traveled differed by colony with linear mixed effects models in the R package lme4 (Bates et al., 2014). Trip statistics were modeled independently as response variables with a Gaussian error distribution, breeding site was included as a covariate, and bird identity was a random effect. Models were compared with null models with only the random effect. We used analysis of variance to test for significance.

Breeding stage at deployment was known at Bird Island and was inferred at Prion Island—where birds were tracked continuously from incubation onward—from trip metrics and colony attendance because foraging trips during incubation and brood‐guard periods are usually followed by >12 h spent at the nest. A bootstrapping approach to assess the representativeness of the samples for each population was undertaken with Track2KBA.

### Spatial segregation

We estimated utilization distributions (UDs) in the core foraging area (50%), home range area (95%) for each population, and the core foraging area for each individual. The smoothing parameter, estimated using track2kba, was consistent within breeding stages and between sites (incubation = 105, brood guard = 60, postguard = 105). The grid cell size was 10 km.

We calculated population‐level overlap in UDs with Bhattacharya's affinity (BA) and used a randomization procedure to test for significant differences in UDs following Clay et al. (2018). The overlap among populations was calculated with BA, and then each trip was randomly assigned to a colony, and new population‐level UDs and BA were calculated. This was repeated 1000 times, and the proportion of occurrences where the randomly assigned overlap was smaller than the overlap from the tracked sample provided the *p* value.

### Influence of wind conditions

To evaluate whether wind speed and direction contributed to spatial segregation, we downloaded from Climate Copernicus the ERA5 wind conditions at the time of departure from a colony (Thépaut et al., 2018). Linear mixed models were used to assess whether wind speed at departure varied between breeding sites. Wind direction at departure at the different breeding sites was compared using Watson's 2‐sample tests of homogeneity. We used the circular package (Lund et al., 2017) to assess whether the direction of the bird at departure (based on mean direction during the first 3 h at sea) and the bearing to the farthest point from the colony were dependent on the direction of the wind at departure (circular‐circular regression) or dependent on wind speed at departure (circular‐linear regression).

### Habitat models

To identify whether spatial segregation may be driven by between‐population variation in preferred foraging habitats, habitat models were developed for each breeding stage and site. All locations were used in the models because wandering albatrosses can forage opportunistically while in directed flight and not just in regions associated with area‐restricted searches (Carneiro et al. 2022a). The latter would in any case be challenging to identify given the interpolated intervals of ∼1 h between PTT fixes. Three pseudoabsences for every location point were generated randomly within the maximum foraging distance of birds from each colony in the R package dismo (Hijmans et al., 2024). Biologically meaningful environmental covariates, including proxies for oceanographic features that enhance prey availability, at appropriate temporal and spatial scales (Appendix S1), were extracted for each location.

To improve the spread of the data, eddy kinetic energy and chlorophyll *a* were transformed to log_10_, and the standard deviation of sea‐surface temperature was transformed to the square‐root (Wood, 2006). General additive mixed models with a binomial error structure and cubic spline smoothing were used to model presence‐absence as a function of environmental covariates; bird identity was included as a random effect. The number of knots was limited to a maximum of 7 to avoid overfitting to the data. Collinearity between covariates was assessed, and where *r* > 0.6, the covariate with the highest predictive power was included in model selection. Final models were checked for concurvity, which was always <0.3. Forward model selection and cross‐validation between individuals were used to develop final models, and the area under the curve (AUC) was used to evaluate predictive power. Values of 0.5–0.7, 0.7–0.9, and >0.9 were considered to represent poor, reasonable, and very good model performance, respectively (Clay et al., 2016). Final models were tested for spatial autocorrelation, which was present in the model residuals. As such, a correlation structure of model residuals was included (following Crase et al., 2012), after which residual spatial autocorrelation was no longer present. Final models were used to predict the distribution of each population across the study area under average environmental conditions for each breeding stage.

### Overlap with fisheries

For each breeding stage (incubation: 9 January−29 March; brood guard: 2 March−18 April, and postguard: 5 April−18 August), fishing activity (total number of hours fished in each breeding stage) across the study area was based on vessel movement tracked with the automatic identification system (AIS). We downloaded these data from Global Fishing Watch at a scale of 1°. The proportion of time that all birds from each colony spent in each 1° grid cell was multiplied by the number of hours fished in that cell to provide an overlap index for each breeding stage (Carneiro et al. 2022b; Frankish et al., 2021). This was calculated for each flag state and each type of fishing that occurred in the study area (drifting [pelagic] longliners, set [demersal] longliners, trawlers, squid jiggers, pole and line, and unknown fishing) and for all fishing combined.

## RESULTS

### Tracking data

Overall, 91 trips and 190 trips were obtained from 85 wandering albatrosses at Bird Island and 10 wandering albatrosses at Prion Island, respectively, in the incubation, brood guard, or postguard periods in January−August 2022 (Figure 1). In total, 28 trips from 28 birds during incubation, 28 trips from 27 birds during the brood‐guard period, and 35 trips from 30 birds in the postguard period were recorded from wandering albatrosses breeding at Bird Island and 25 trips from 10 birds during incubation, 45 trips from 10 birds during the brood‐guard period, and 120 trips from 10 birds during the postguard period were recorded from wandering albatrosses breeding at Prion Island. These included both complete and incomplete trips. Tracks provided a good representation of the distribution of each population: Bird Island, incubation 91%, brood‐guard period 93%, postguard period 95%, and Prion Island, incubation 94%, brood‐guard period 96%, postguard period 99% (Appendix S2) These values are the percentage of the core area used by these populations that was captured in our sample. These were considered sufficient to make population‐level inferences; however, we may not have included the entire extent of the population range for each population. The mean and frequency distributions of trip duration, total travel distance, and maximum range were not statistically different between the 2 breeding sites during any of the 3 breeding stages (Table 1 & Appendices S3 and S4).

### Spatial segregation

During incubation, birds from Bird Island traveled west to the North Scotia Arc, southern Argentine Basin, and Patagonian Shelf break and southwest to the Antarctic Peninsula and through the Drake Passage into the southeastern Pacific Ocean. In contrast, although the birds on Prion Island used some of those westerly destinations, they rarely went southwest (i.e., to the Antarctic Peninsula and southeastern Pacific). Instead, they traveled east to as far as the Mid‐Atlantic Ridge and South America‐Antarctic Ridge (Figure 1). During incubation, the home range but not the core foraging areas of the 2 populations were more segregated than expected by chance (Appendix S5). During the brood‐guard period, although both populations remained close to South Georgia, spatial segregation was observed in the core foraging and home range areas. During the postguard period, both populations predominantly traveled west to waters from the Burdwood Bank north to the Subtropics and throughout the Argentine Basin. There was significant spatial segregation in home range but not in core foraging areas. Based on the core foraging areas of individuals, almost all areas specific to each population were used by multiple birds (Appendix S6).

### Influence of wind conditions

Wandering albatrosses from Bird Island and Prion Island all experienced predominantly southwesterly winds during all phases of the breeding season (wind direction for each population, respectively and Watson's 2‐sample test for homogeneity: incubation mean [SD] = 226° [60], 234° [42], *U*_2_ = 0.05, *p* > 0.10; brood‐guard period mean = 226° [85], 245° [55], *U*_2_ = 0.17, *p* > 0.10; postguard period mean = 270° [66], 255° [56], *U*_2_ = 0.11, *p* > 0.10). During incubation and brood‐guard periods, birds from both breeding sites experienced winds of similar strength, whereas during the postguard period, birds at Prion Island experienced lighter winds than those at Bird Island (χ^2^ = 8.82, *p* = 0.04) (Appendix S7). We found no association between the wind direction and either the bearing of the bird in the first 3 h or the bearing of the point farthest from the colony across breeding stages, with the exception that the latter was significantly related to wind direction for birds from Bird Island during the postguard period (cos(*x*) *p* = 0.03, sin(*x*) *p* = 0.73). During this period, the direction of travel in the first 3 h and bearing to the point farthest from the colony were associated with wind speed at departure for birds from both breeding sites (Appendix S7). For trips from Prion Island, where the bearing to the farthest point was southeast, wind speed at departure was always light (Appendices S8−S10). However, the sample size was too low to draw conclusions as to whether birds only travel southeast when leaving the colony in light winds.

### Habitat models

**FIGURE 2:**
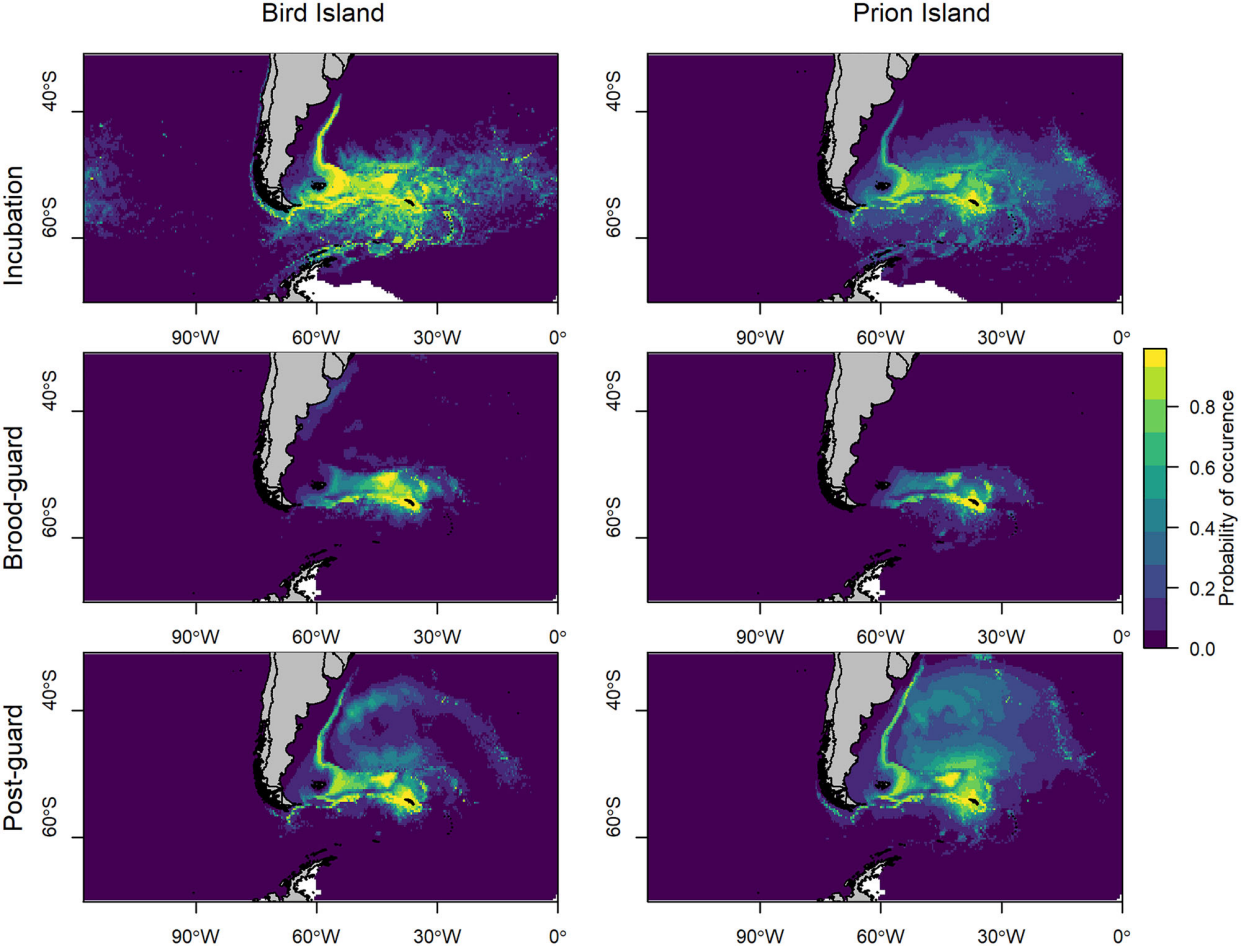
Predicted probability of occurrence of wandering albatrosses breeding at Bird Island and Prion Island, South Georgia, for each stage of the 2021−2022 breeding season.

Habitat models for both colonies had high predictive power and deviance explained with an AUC range of 0.8−0.93 and a deviance explained range of 0.42−0.61 (Appendix S11). For each site and stage of the breeding season, distance to colony, sea surface temperature, and depth were included in the final models. Including eddy kinetic energy increased the predictive performance of models for both colonies during incubation. Birds from both colonies tended to prefer areas close to the colony, water temperatures of 0−20°C, and depths of <1 km. A second peak in depth at approximately 6−8 km may have reflected the cluster of locations in the southern Argentine Basin (Appendices S12−S14). Predicted distributions from final models were similar to observed distributions (Figure 2). Based on model predictions, habitat conditions to the east and west of South Georgia, and at the Antarctic Peninsula, were suitable for birds from both Prion Island and Bird Island in incubation, yet our tracking data showed no birds from Bird Island traveled east and no birds from Prion Island traveled to the Antarctic Peninsula.

### Overlap with fisheries

**FIGURE 3:**
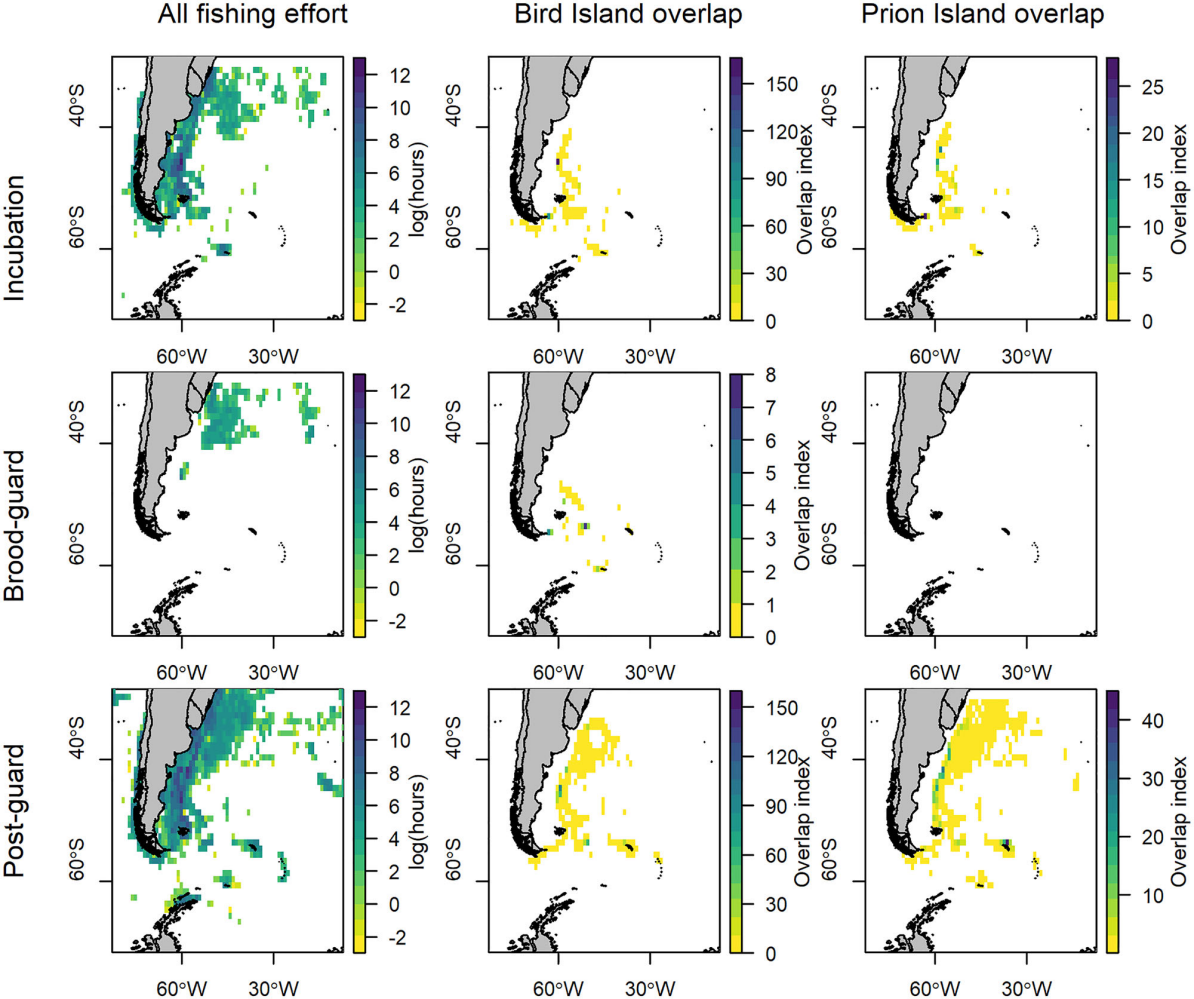
Fishing effort (log of total hours of fishing in each cell) and overlap of fishing with wandering albatrosses breeding at Bird Island and Prion Island, South Georgia, during incubation (9 January−29 March), brood guard (2 March−18 April), and postguard (5 April−18 August) periods of the 2022 breeding season. Appendix S15 contains effort and overlap by gear type. Overlap indices have different scales.

**FIGURE 4:**
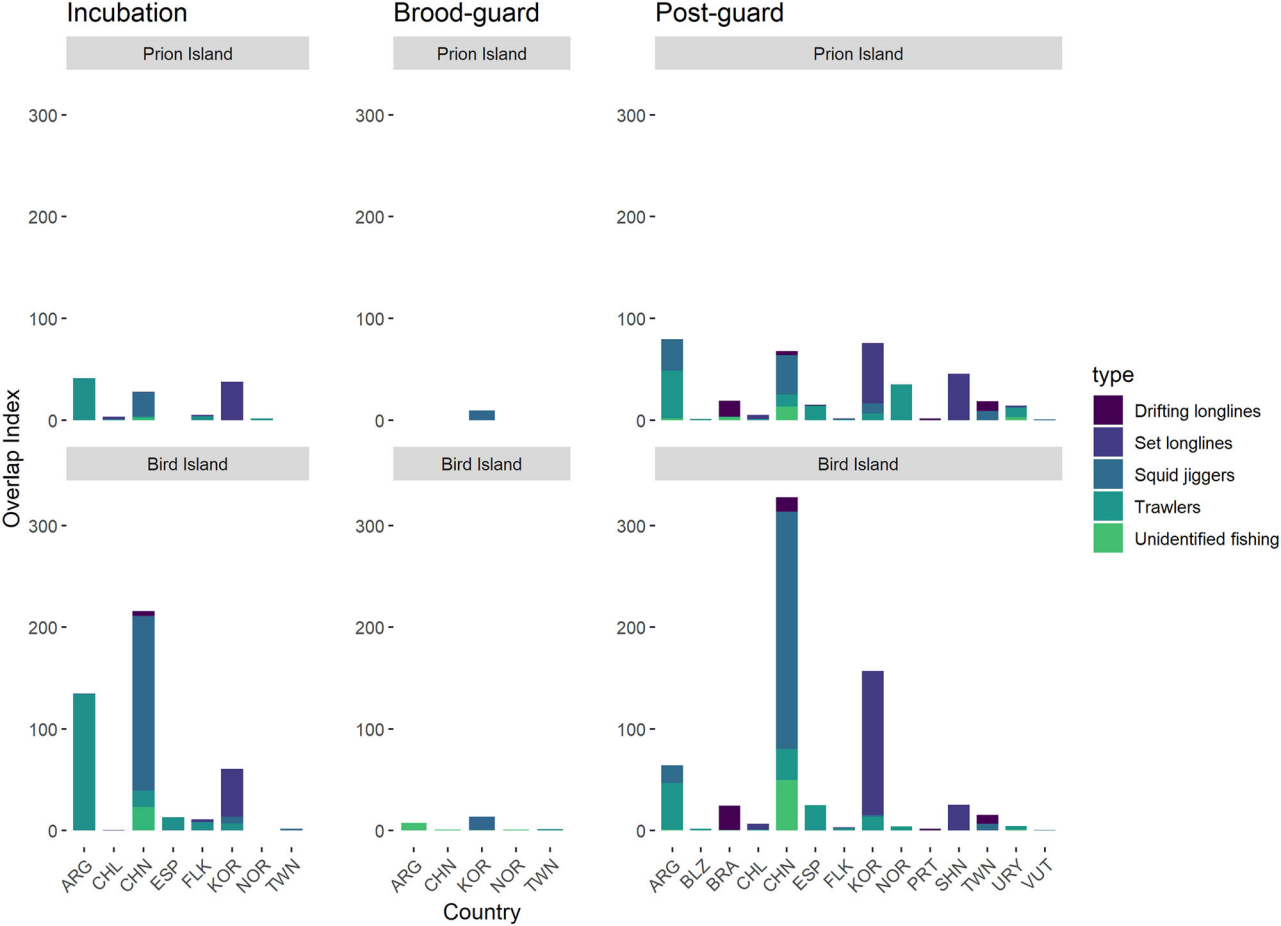
Overlap of wandering albatross breeding at Bird Island and Prion Island, South Georgia, during incubation, brood guard, and postguard periods in the 2022 breeding season with fishing vessels by flag state (abbreviations) and gear type (shading, primary fishing gear type for vessels actively fishing; ARG, Argentina; CHL, Chile; CHN, China; ESP, Spain; FLK, Falkland Islands; KOR, South Korea; NOR, Norway; TWN, Taiwan; BLZ, Believe; BRA, Brazil; PRT, Portugal; SHN, Saint Helena; Ascension and Tristan da Cunha; URY, Uruguay; VUT, Vanuatu).

Overlap with all types of fisheries occurred predominantly at the Patagonian Shelf break and farther offshore in the Argentine Basin, at the Subtropical Convergence, or around South Georgia and the South Orkney Islands during the postguard period (Figure 3). The fisheries overlap index was considerably higher for wandering albatrosses from Bird Island than Prion Island during all stages of the breeding season for all fishing and in almost all cases when overlap was calculated for each gear type, which included drifting (pelagic) longline, pole and line, set (demersal) longline and trawl (Table 2 & Appendix S15). The exception was during postguard period, when there was greater overlap with squid jiggers and trawlers for birds from Prion Island than Bird Island. During incubation, overlap between wandering albatrosses and drifting (pelagic) longlines and unknown fishing was 5 times higher for birds from Bird Island than those from Prion Island, although these values were relatively low relative to overlap with other gear types. Overlap with trawlers was nearly 4 times higher for birds from Bird Island than Prion Island, and the overlap index was relatively high for birds from both colonies. Birds from both colonies overlapped most frequently with Chinese squid jiggers, Argentinian trawlers, and South Korean set (demersal) longliners (Figure 4).

**TABLE 2:** Index values for the overlap between wandering albatrosses breeding at Bird Island and Prion Island, South Georgia, during the 2021−2022 breeding season and fisheries operating within their foraging range.*

	Prion Island				Bird Island			
	incubation	brood guard	postguard	total	incubation	brood guard	postguard	total
All fishing	97	9	380	486	335	25	551	911
Drifting (pelagic) longlines	0.3	0	34	34.3	1.5	0	43	44.5
Unknown fishing	4	0	27	31	20	0	84	104
Pole and line	0	0	2	2	0.1	0	6	6.1
Set (demersal) longlines	29	9	98	136	39	12	162	213
Squid jiggers	37	0	208	245	45	0	188	233
Trawlers	44	0	177	221	152	8	144	304

* Duration differs among incubation (9 January−29 March), brood guard (2 March−18 April), and postguard (5 April−18 August) periods, so the overlap index values can be compared among sites but not among breeding stages.

## DISCUSSION

Many studies have reported spatial segregation between colonies of wide‐ranging species situated hundreds of kilometers apart (Ramos et al., 2013; Wakefield et al., 2013) and in species with smaller foraging ranges breeding in colonies situated close together (Bertrand et al., 2021; Wakefield et al., 2017; Wanless & Harris, 1993). The few relevant studies of wide‐ranging species in closely situated colonies have provided mixed evidence for spatial segregation (Ceia et al., 2015; Pereira et al., 2022; Waggitt et al., 2014). By contrast, we found an unexpectedly high degree of spatial segregation in the at‐sea distributions of wandering albatrosses breeding at Bird Island and Prion Island. Although birds from both colonies foraged predominantly on the North Scotia Arc, southern Argentine Basin, and shelf break of the Patagonian Shelf, only those from Prion Island frequently traveled east of South Georgia to as far as the Mid‐Atlantic Ridge and South America‐Antarctic Ridge, and only those from Bird Island to the Antarctic Peninsula and southeast Pacific Ocean. The spatial segregation had major implications for overlap with fisheries and, hence, potentially bycatch risk, population dynamics, and conservation.

It is important in general to understand spatial segregation between colonies because tracking studies are subject to financial and logistical constraints. Thus, information about distribution from one site is frequently used to make inferences about that species in the entire island group. However, the assumption that foraging distributions are homogenous could bias conclusions about relative risk from at‐sea threats and lead to suboptimal allocation of resources for monitoring or management. To our knowledge, ours is the first study to show that spatial segregation and differing fisheries overlap between closely situated colonies of a wide‐ranging seabird are the most probable drivers of contrasting population trends. Additional discussion about the potential mechanisms driving spatial segregation is in Appendix S16.

### Competition

Spatial segregation in the foraging distribution of birds from different colonies may be a response to density‐dependent competition in central‐place foragers (Bolton et al., 2019; Wakefield et al., 2013; Wakefield et al., 2017). We found high overlap on the Patagonian Shelf break, near the limit of the foraging range of birds from both colonies, which is consistent with the idea that areas of high productivity farther from the colony enable higher overlap. However, overlap along the North Scotia Arc and the Burdwood bank was also high, suggesting that mechanisms other than density‐dependent competition are involved. Indeed, density‐dependent competition does not explain why only birds breeding on Prion Island traveled east and only birds breeding at Bird Island traveled to the Antarctic Peninsula and farther west to feed.

### Influence of wind

Trip metrics were similar at the 2 colonies, suggesting that the differences in foraging distributions were not a consequence of habitat accessibility or energetics. However, the observed spatial segregation could be a consequence of wind conditions because wind strength and direction affect the flight and foraging behavior of many pelagic seabirds (Spear & Ainley, 1997; Ventura et al., 2020; Weimerskirch et al., 2000) and can influence the bearing of departure of birds from the colony (Clay et al., 2023; Grémillet et al., 2004). However, wandering albatrosses from both sites experienced predominantly southwesterly winds of similar strength at the time of departure; thus, it is unlikely that the observed spatial segregation is a result of variation in localized wind conditions at the colony.

### Habitat specialization

Habitat specialization has the potential to drive spatial segregation if the preference for different habitat characteristics leads to foraging in different locations. Our habitat models identified very similar habitat preferences, indicative of areas of high productivity, for birds breeding at Prion Island and Bird Island (Scales et al., 2016; Wakefield et al., 2011; Warwick‐Evans et al., 2021). Our models indicated that in areas where segregation was observed, both east and west of South Georgia habitat characteristics were suitable for birds from both colonies. It was not clear why these were visited by individuals from just 1 colony. Regardless, given that habitat preferences for birds from both colonies are very similar, there is little indication that habitat specialization drives spatial segregation within this island group.

### Information sharing, memory, and cultural evolution

In the absence of a convincing alternative, another explanation for spatial segregation is social learning and past experiences of each individual because information exchanged at the colony may result in the cultural evolution of foraging patterns (Morinay et al., 2023; Wakefield et al., 2013). Indeed, one of the benefits of colonial breeding is the transfer of information among individuals, which may facilitate group foraging (Grémillet et al., 2004; Sutton et al., 2017; Wakefield et al., 2013; Weimerskirch et al., 2010). Alternatively, memorized personal information alone may lead to spatial segregation between colonies, independent of the transfer of social information (Aarts et al., 2021). However, that seems less likely in our study because many wandering albatrosses remain in the southwest Atlantic from fledging until they first return to the colony, when they feed over very large areas that potentially encompass most or all of those used by breeding adults from both Bird Island and Prion Island. As such, it is not clear why birds from Bird Island would not continue to use the habitat east of South Georgia even after recruitment. Instead, we suggest that cultural foraging patterns may have emerged that differed between the 2 populations. Young, naïve birds follow more experienced adults in other species (Bolton et al., 2019; Votier et al. 2011; Ward and Zahavi 1973). The recruitment process takes several years, with individual wandering albatrosses arriving earlier and spending increasing time at the colony until they breed for the first time (Pickering 1989). It is, therefore, possible that in those formative years, when immatures are constrained to forage from a central place, they develop a more restricted range, potentially following established breeders to areas specific to that colony. Although this remains speculative in the absence of further study, it is perhaps the most plausible explanation for the segregation of foraging areas that we observed.

### Overlap with fisheries

The spatial segregation we observed affected potential fisheries bycatch risk, given the higher overlap of birds from Bird Island than those from Prion Island, with almost all fishing types during all breeding stages. As such, relative bycatch rate is likely to be a key driver of the contrasting population trends, although other factors may also be involved. Variability in rates of decline between seabird populations can be due to differences in resource availability or quality, competition, disease, chick or egg predation at the colony, prey availability, or threats encountered during the nonbreeding period or other life‐history stages (Bogdanova et al., 2017; Frederiksen et al., 2005; Kitaysky et al., 2010; Lewis et al., 2006). However, most of these seem unlikely to apply. One might expect that differences in resources or competition at sea would affect foraging success during the breeding season, yet there were no differences in trip summary statistics, such as trip duration, which may increase when foraging efficiency is low (Chivers et al., 2012; Lorentsen et al., 2019). Nor are there any land‐based predators at Bird Island or Prion Island. Variability in prey availability and threats encountered during the nonbreeding season may be contributing to population declines and warrant further investigation (Clay et al., 2019; Whitehouse et al., 2012). Regardless, the clear contrast between the 2 sites in relative overlap with fisheries during the breeding season seems highly likely to be one of the main drivers of variation in rates of population decline, given that bycatch in fisheries is one of the most serious threats to this species and to pelagic seabirds in general (Pardo et al., 2017; Phillips et al., 2016; Tuck et al., 2003).

Overlap with fisheries was higher during incubation and postguard periods for both Bird Island and Prion Island. During these periods, foraging trips were longer and birds traveled from the South Georgia Shelf west to the Burdwood Bank, Patagonian shelf break, and pelagic waters farther offshore, including around the Subtropical Convergence. These areas of greatest overlap were consistent with many previous studies showing high albatross‐fisheries overlap on the Patagonian Shelf and the Brazil‐Falklands confluence (Carneiro et al. 2022b; Clay et al., 2019; Jiménez et al., 2014).

The highest overlap for wandering albatrosses from Prion Island and Bird Island was with Chinese squid jiggers, Argentinian trawlers, and South Korean set (demersal) longliners. Wandering albatrosses encounter and visit all of these vessel types (Carneiro et al. 2022b), but apparently they are not frequent bycatch of trawlers and squid jiggers based on available observer data (Adasme et al., 2019; FIG, 2021; Reid et al., 2021). Bycatch rates on demersal longliners on the Patagonian Shelf also seem to be low (Favero et al., 2013), but their high susceptibility in demersal fisheries elsewhere prior to the adoption of effective bycatch mitigation indicates that the risk is not trivial (Collins et al., 2021). Bycatch rates of wandering albatrosses are highest in drifting (pelagic) longline fisheries because birds attempting to feed on the baited hooks are caught and drowned (Jiménez et al., 2014, 2016). We found that overlap with drifting (pelagic) longlines was low in comparison with other gear types, although during the postguard period, birds from both breeding sites overlapped with the fleets of Brazil, China, and Taiwan. Results of previous studies show low overlap and encounter rates of wandering albatrosses with drifting (pelagic) longliners, particularly during austral summer (Clay et al., 2019; Carneiro et al. 2022b), but authors conclude that although these interactions may be infrequent, the risk during encounters must be considerably higher than those posed by other vessel types to result in high observed bycatch rates (Jiménez et al., 2014, 2016).

Implementing mitigation measures, including bird‐scaring lines, night setting, and heavier line‐weighting, can greatly reduce seabird bycatch rates in demersal and pelagic longline fisheries (Collins et al., 2021; Da Rocha et al., 2021; Phillips et al., 2016). The Agreement for the Conservation of Albatrosses and Petrels (ACAP) advises that the simultaneous use of 3 mitigation measures is best practice in mitigating seabird bycatch (ACAP, 2024). However, mitigation requirements within Exclusive Economic Zones (EEZs) are set and enforced by the relevant territory and in the high seas by Regional Fisheries Management Organisations (RFMOs). Differences in mitigation requirements and the monitoring of compliance between RFMOs and other bodies will affect bycatch rates, particularly because compliance with conservation measures is low in the absence of observers (Gilman, 2011). For example, in the southwestern Atlantic Ocean, the pelagic longline fisheries are regulated by the International Commission for the Conservation of Atlantic Tunas (ICCAT), which requires 2 mitigation measures in waters south of 25⁰ S and 1 measure between 20⁰ S and 25⁰ S. Observer coverage in the ICAAT fleets is <5% of the fishing effort (Jiménez et al., 2020), and bycatch rates remain relatively high. In contrast, the Committee for the Conservation of Antarctic Marine living resources (CCAMLR), which regulates demersal longline fishing around subantarctic islands (including South Georgia) and in the Ross Sea, requires multiple mitigation measures, including night setting, line weighting, streamer lines, thawed bait, bird exclusion devices, ban on offal discharge during fishing activity, seasonal closures, and 100% observer coverage (Collins et al., 2021). This has led to a virtual elimination of seabird bycatch in the South Georgia fishery for Patagonian toothfish (*Dissostichus eleginoides*) (Collins et al., 2021). However, the continued population declines of albatrosses at South Georgia indicate that bycatch mitigation measures in areas of overlap with fisheries away from South Georgia are inadequate (Clay et al., 2019; Jiménez et al., 2014; Pardo et al., 2017).

Although ICAAT and CCAMLR regulate the pelagic longline fishery in areas beyond national jurisdiction (ABNJ) and the demersal longline and pelagic trawl fisheries south of the Antarctic Polar Front, respectively, within the foraging range of wandering albatrosses breeding at South Georgia, currently, no RFMO regulates the other demersal fisheries in this region (Bell et al., 2019). However, the overlap of wandering albatrosses from both our study sites with trawlers and demersal (set) longliners on the Patagonian Shelf outside the EEZs of Argentina and the Falkland Islands was high. Even though these vessels are, in theory, under the jurisdiction of their flag states, the lack of an RFMO makes it unlikely that bycatch mitigation measures are in use or that observers are on board to monitor seabird bycatch, at least for the distant‐water fleets. As such, bycatch rates in these demersal (set) longline and trawl fisheries in ABNJs remain unknown but could include large numbers of wandering albatrosses from South Georgia. Similarly, although national fisheries bodies regulate fishing within their EEZs, mitigation requirements are highly variable (Baker et al., 2024). For example, trawlers operating in Argentina are only required to implement 1 mitigation measure, whereas pelagic longliners operating in the Brazilian EEZ, in theory, must implement 3 measures (Baker et al., 2024).

Many artisanal and small inshore fishing vessels operating within the Brazilian EEZ do not have AIS (Bugoni et al., 2008). We are mindful that the Global Fishing Watch (GFW) dataset does not encompass all fishing activity in some regions where satellite coverage is poor, small vessels (and some large vessels) lack AIS, and some operators disable AIS. In these situations, illegal, unreported, and unregulated fishing is, therefore, excluded (Carneiro et al. 2022b; Frankish et al., 2020; Orben et al., 2021). However, GFW provides the most comprehensive data on fishing effort available in our study area for recent years, and it is likely that any biases would be similar for birds from both colonies.

The overlap index provides a measure of time spent in areas where fisheries operate, but is not a direct measure of bycatch rates. Individual birds may respond differently to vessels (attraction or avoidance behavior [Collet et al., 2017]), and not all wandering albatrosses switch to scavenging when encountering a vessel (Carneiro et al. 2022b). Nevertheless, although bird capture rates vary extensively between vessels and fleets (Jiménez et al., 2014), regions of high seabird‐vessel overlap tend to correspond well with documented bycatch rates (Clay et al., 2019; Jiménez et al., 2016).

### Implications for management

By improving understanding of the distributions of wandering albatrosses from different colonies, and consequently the risks from different fishing fleets, management efforts can be better focused for this ACAP high‐priority population. This includes an urgent need to regulate fisheries in currently unregulated areas, in addition to improving mitigation and observer coverage within EEZs and RFMOs. Tougher mitigation requirements require engagement with RFMOs, fisheries operators, and crew from relevant nations to ensure mandatory implementation of best‐practice seabird bycatch mitigation and monitoring of compliance (Clay et al., 2019; Phillips, 2013).

## AUTHOR CONTRIBUTIONS

R.A.P. and V.W.‐E. designed the project. V.W.‐E., R.A.P., and L.T. acquired the funding and planned the fieldwork. V.W.‐E. and E.J.P. carried out the fieldwork. V.W.‐E. wrote the original draft. All authors reviewed and edited the text.

## Supporting information



Supporting information
