# Supplementary material for: Spatial segregation and bycatch risk as potential drivers of population trends of wandering albatrosses at South Georgia
Source: Conserv Biol. 2025 Aug 20;40(1):e70126. doi: 10.1111/cobi.70126 (PMC12856804; doi:10.1111/cobi.70126)
Supplement: Supplementary file 1 — Supporting information [file COBI-40-e70126-s001.docx]

Title: Spatial segregation and bycatch risk as potential drivers of population trends of wandering albatrosses at South Georgia

**Appendix**

Table S1. Environmental covariates used in model selection for wandering albatrosses breeding at South Georgia. Daily values for all dynamic variables were downloaded and evaluated.

| Predictor | Resolution | Description | Source |
| --- | --- | --- | --- |
| Distance to colony (km) | 0.3 km | Describes accessibility | Calculated using gridDistance function in R package *raster* |
| Depth (km) | 0.3 km | Topographic features (eg seamounts and canyons) may aggregate prey | (Dickens et al. 2014) |
| Slope (degrees) | 0.3 km | Steep slopes may aggregate prey, or shallow plateaus may retain prey, or increase prey availability | Calculated from depth in R |
| Mean sea level anomaly (MSLA) (m) | 0.25 degree | Frequently used to measure eddies and fronts which may aggregate prey | http://marine.copernicus.eu/ |
| Sea Surface Temperature (SST)  (degrees C) | 0.083 degree | Frequently used to reflect optimal conditions for prey items | <http://marine.copernicus.eu/>  Global_Reanalysis_Phy_001_030 |
| Sea Surface Temperature Standard Deviation (SSTsd) (degrees C) | 0.083 degree | Frequently used to reflect optimal conditions for prey items | Calculated in R from SST |
| Eddy Kinetic Energy (EKE)(m^2^ s^2^) | 0.083 degree | Frequently used to measure eddies and fronts which may aggregate prey | <http://marine.copernicus.eu/>  Global_Reanalysis_Phy_001_030 |
| Chlorophyll-a mass concentration (CHL) | 4 km | Frequently used as a proxy for prey availability | <http://marine.copernicus.eu/>  Global Ocean Chlorophyll, PP and PFT (Copernicus-GlobColour) from Satellite Observations: Monthly and Daily Interpolated (Reprocessed from 1997) |

**
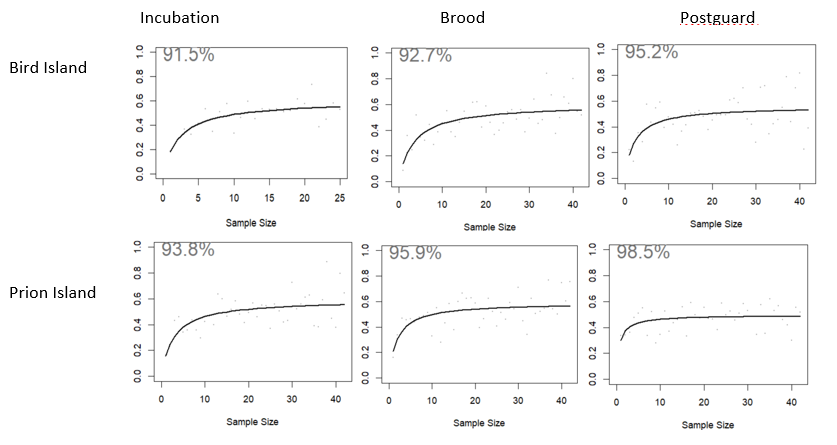
**

Figure S2. Plots of the asymptotes used in the bootstrapping approach to measure how representative of the entire population our sample of tracks is, where the value stated is the percentage of the core area used by these populations that are captured in our sample


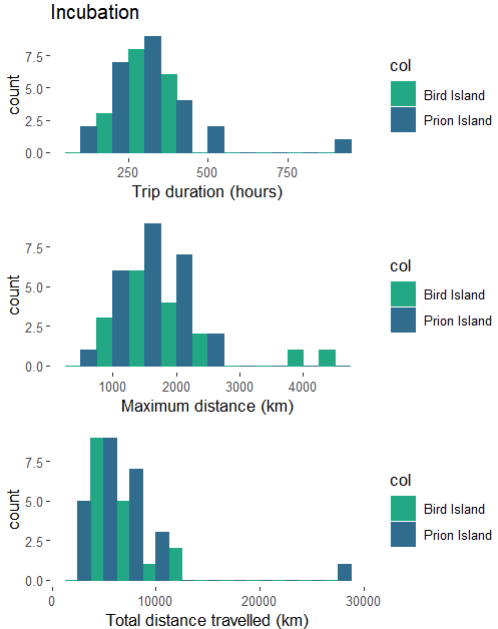

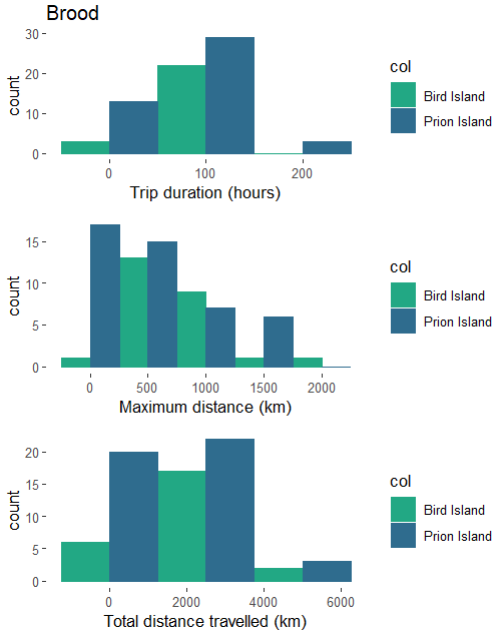


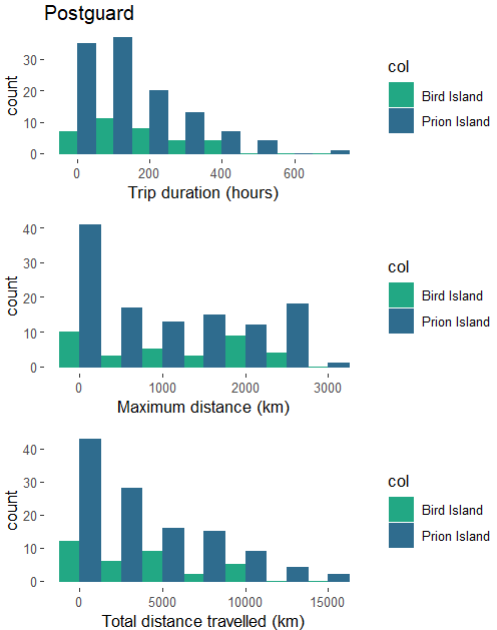


Figure S3. Trip summary statistics for wandering albatrosses tracked from Bird Island and Prion Island, South Georgia during the 2022 breeding season.

Table S4. linear mixed models comparing the trip summary statistics of wandering albatrosses tracked from Bird Island and Prion Island, South Georgia during the 2021/2022 breeding season

| Stage | Variable | Chi sq | Df | P |
| --- | --- | --- | --- | --- |
| Incubation | Trip duration | 0.319 | 1 | 0.57 |
|  | Total distance travelled | 0.72 | 1 | 0.40 |
|  | Foraging range | 1.6 | 1 | 0.2 |
|  | Wind speed at departure | 0.73 | 1 | 0.39 |
| Brood-guard | Trip duration | 0.06 | 1 | 0.79 |
|  | Total distance travelled | 0.67 | 1 | 0.41 |
|  | Foraging range | 2.96 | 1 | 0.09 |
|  | Wind speed at departure | 0.7 | 1 | 0.40 |
| Post-guard | Trip duration | 0.25 | 1 | 0.61 |
|  | Total distance travelled | <0.001 | 1 | 0.99 |
|  | Foraging range | 0.62 | 1 | 0.43 |
|  | Wind speed at departure | 8.25 | 1 | 0.004 |

Table S5**.** Observed and randomised overlap (Bhattacharya’s Affinity) of Utilisation Distributions (UDs) of wandering albatrosses tracked from Bird Island and Prion Island, South Georgia, during the 2021/22 breeding season.

| Utilisation Distribution | Stage | Sample overlap | Randomised overlap | *P* |
| --- | --- | --- | --- | --- |
| 50% (Core foraging area) | Incubation | 0.35 | 0.38 ± 0.03 | 0.17 |
|  | Brood-guard | 0.27 | 0.44 ± 0.02 | **< 0.001** |
|  | Post-guard | 0.36 | 0.40 ± 0.02 | 0.09 |
| 95% (Home Range Area) | Incubation | 0.69 | 0.77 ± 0.03 | **0.005** |
|  | Brood-guard | 0.74 | 0.82 ± 0.02 | **< 0.001** |
|  | Post-guard | 0.78 | 0.85 ± 0.02 | **0.02** |

Footnotes: Randomized overlaps are shown as a mean ± SD and *P* represents the proportion of randomized overlaps that were smaller than the observed overlap; significant values are in bold. Maximum values for Core Foraging Area are 0.5 and for Home Range Area are 0.95.


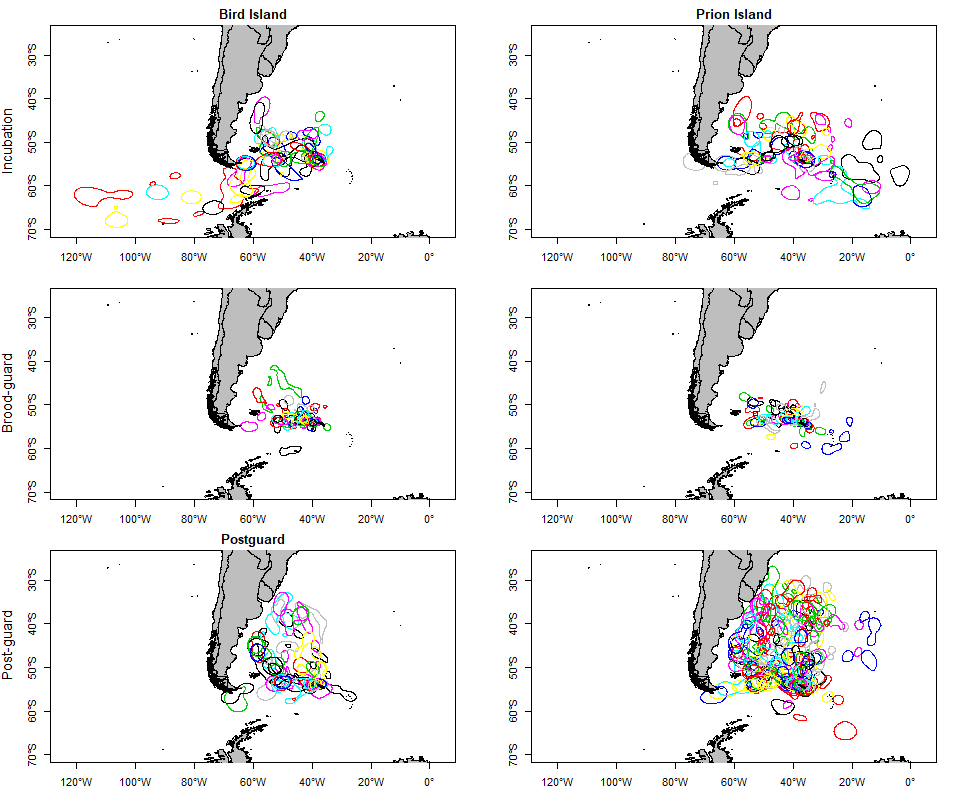


Figure S6. Utilisation Distributions describing the Core Foraging Area (50 %) of individual wandering albatrosses tracked from Bird Island and Prion Island during the 2022 breeding season.

Table S7. Circular-linear models testing whether wind speed effects the direction at departure (first 3 hours) or the bearing to the point at the maximum distance from the colony of wandering albatrosses tracked from Bird Island and Prion Island, South Georgia during the 2021/2022 breeding season. Significant results from t-statistics are highlighted in bold.

| Stage | colony | Model | t-value | P |
| --- | --- | --- | --- | --- |
| Incubation | Bird Island | Bird direction departure~windspeed | 1.3 | 0.11 |
|  | Bird Island | Bird direction maxpoint~windspeed | 1.28 | 0.12 |
|  | Prion Island | Bird direction departure~windspeed | 1.74 | 0.07 |
|  | Prion Island | Bird direction maxpoint ~windspeed | 1.56 | 0.08 |
| Brood-guard | Bird Island | Bird direction departure~windspeed | 1.32 | 0.1 |
|  | Bird Island | Bird direction maxpoint ~windspeed | 1.44 | 0.09 |
|  | Prion Island | Bird direction departure~windspeed | 1.34 | 0.1 |
|  | Prion Island | Bird direction maxpoint ~windspeed | 1.52 | 0.08 |
| Post-guard | Bird Island | Bird direction departure~windspeed | 5.81 | **<0.01** |
|  | Bird Island | Bird direction maxpoint ~windspeed | 3.42 | **<0.01** |
|  | Prion Island | Bird direction departure~windspeed | 3.97 | **<0.01** |
|  | Prion Island | Bird direction maxpoint ~windspeed | 3.55 | **<0.01** |


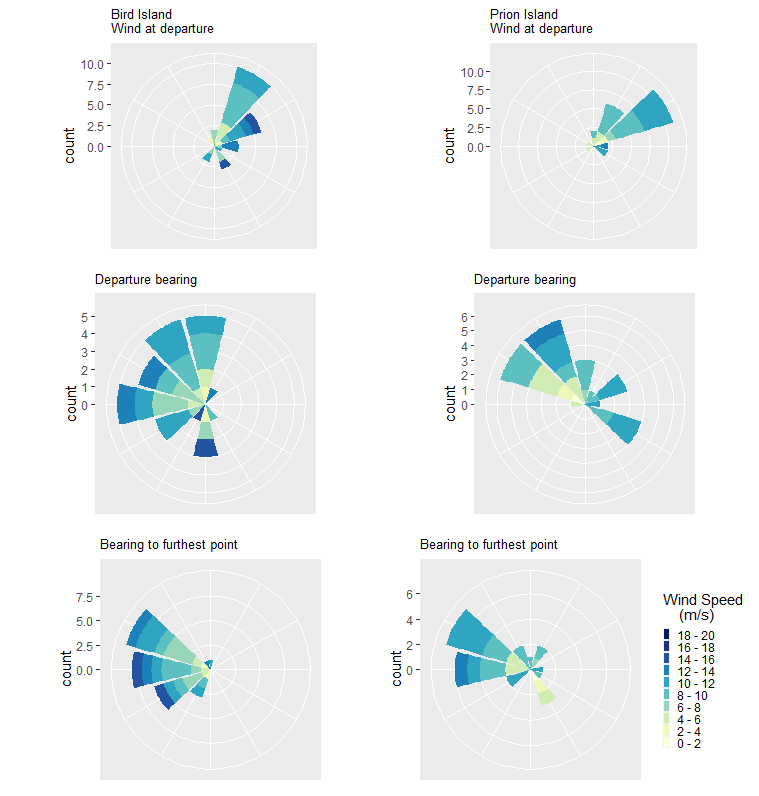


**Figure S8.** Wind conditions at the time of departure from the colony for wandering albatrosses tracked on foraging trips in incubation from Bird Island and Prion Island, South Georgia during the 2021/2022 breeding season. Panels indicate; wind direction and speed at the time of departure from the colony, bearing of departure from the colony with wind speed, and bearing from the colony to the furthest point with wind speed. For consistency, both wind direction and bird direction are shown as the direction they are heading towards (not coming from). The wind speed is at the time the bird leaves the colony. See appendix for wind conditions during brood-guard and post-guard.


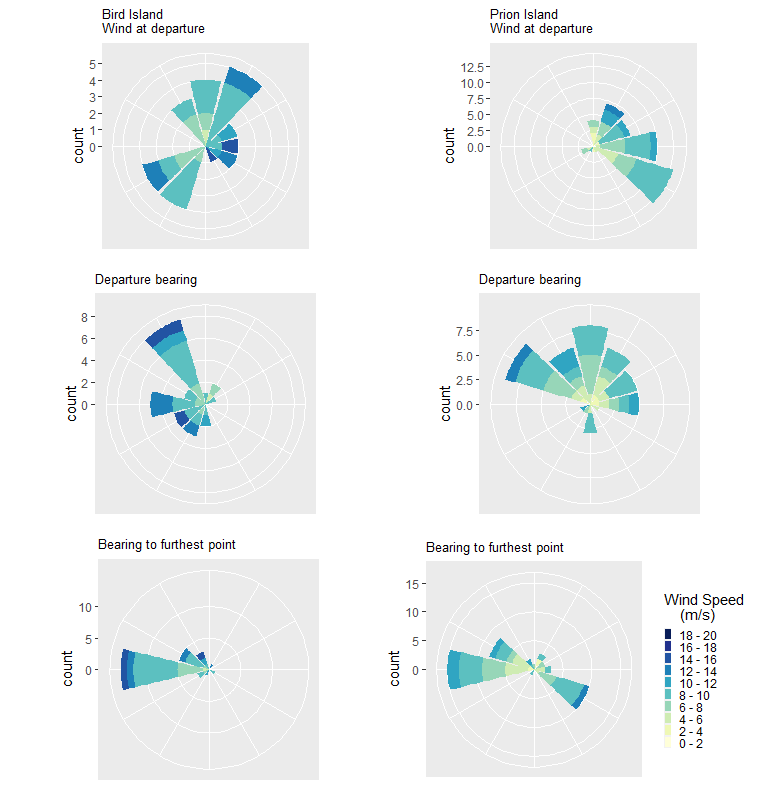


Figure S9. Wind direction and speed at time of departure from the colony, bearing of departure from the colony and bearing from the colony to the furthest point for each trip recorded during the brood-guard stage of the 2022 breeding season. Both wind direction and bird direction are shown as the direction they are heading towards (not coming from). The wind speed on all plots is at the time the bird leaves the colony.


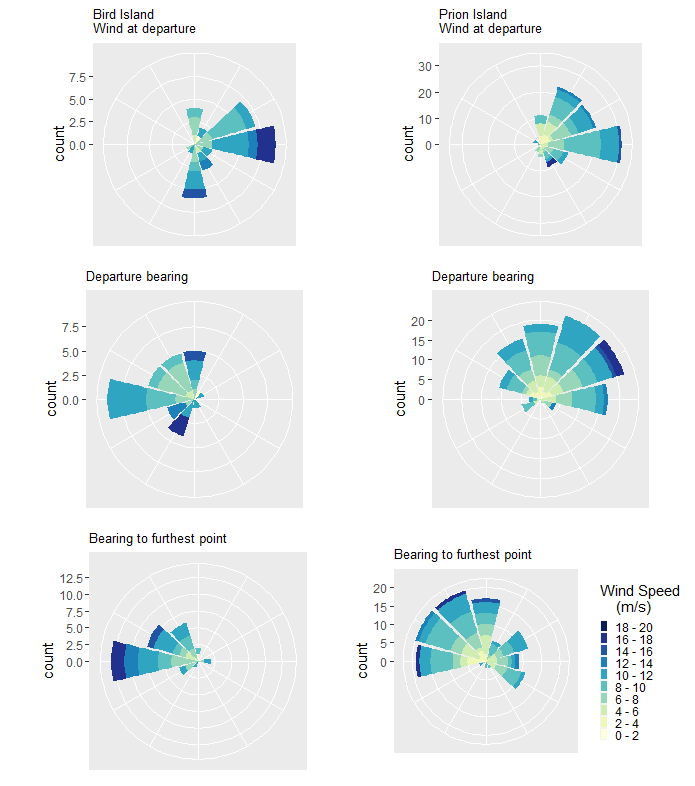


Figure S10. Wind direction and speed at time of departure from the colony, bearing of departure from the colony and bearing from the colony to the furthest point for each trip recorded during the post-guard stage of the 2022 breeding season. Both wind direction and bird direction are shown as the direction they are heading towards (not coming from). The wind speed on all plots is at the time the bird leaves the colony.

Table S11. Covariates in models predicting the distribution of wandering albatrosses breeding at Bird Island and Prion Island, South Georgia, during the 2021/22 breeding season.

| Colony | Stage | Model | Area Under Curve | Deviance Explained |
| --- | --- | --- | --- | --- |
| Bird Island | Incubation | s(distcol, k=4) +s(depth, k=7)+s(sst, k=5)+s(log_eke, k=5) | 0.93 | 61 |
|  | Brood-guard | s(distcol, k=4) + s(sst, k=5)+ s(depth, k=7) | 0.92 | 59 |
|  | Post-guard | s(distcol, k=6) + s(sst, k=6)+ s(depth, k=6) | 0.91 | 50 |
| Prion Island | Incubation | s(sst, k=5)+ s(depth, k=5)+ s(distcol, k=6) + s(log_eke, k=6) | 0.80 | 42 |
|  | Brood-guard | s(depth, k=5)+ s(distcol, k=4) + s(sst, k=6) | 0.90 | 54 |
|  | Post-guard | s(distcol, k=6) +s(depth, k=5)+s(sst, k=6) | 0.87 | 45 |

footnotes: Covariates are shown in order of model selection (forwards) and were evaluated via cross-validation between individuals. A covariate of model residuals was included in each model to account for spatial autocorrelation, and bird ID was included as a random effect. Covariates include: distance to breeding colony (distcol), depth (depth), sea surface temperature (sst), and eddy kinetic energy (log_10_ transformed, log_eke).


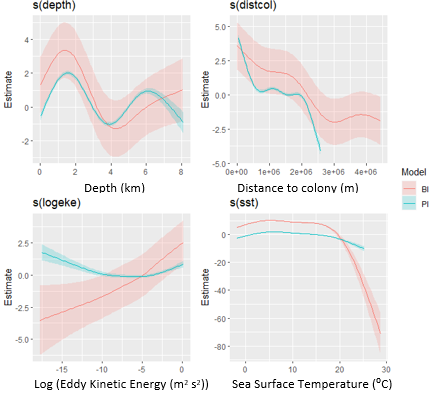


Figure S12. Response curves of models describing the distribution of wandering albatrosses breeding at Bird Island (BI) and Prion Island (PI), South Georgia during incubation.


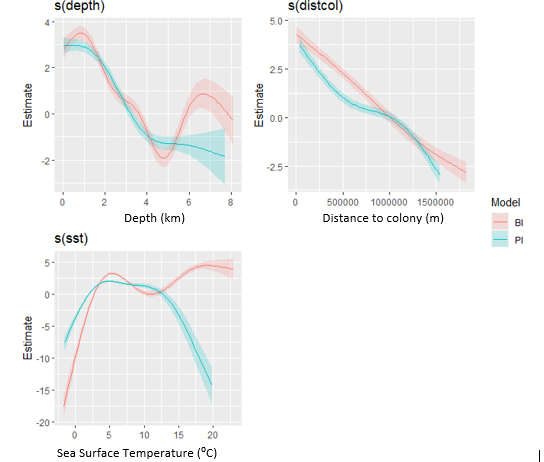


Figure S13. Response curves of models describing the distribution of wandering albatrosses breeding at Bird Island and Prion Island, South Georgia during brood-guard.


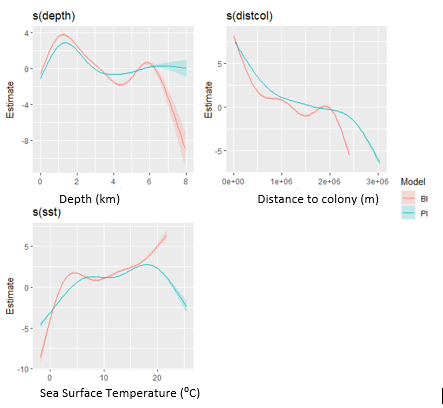


Figure S14. Response curves of models describing the distribution of wandering albatrosses breeding at Bird Island and Prion Island, South Georgia during post-guard.


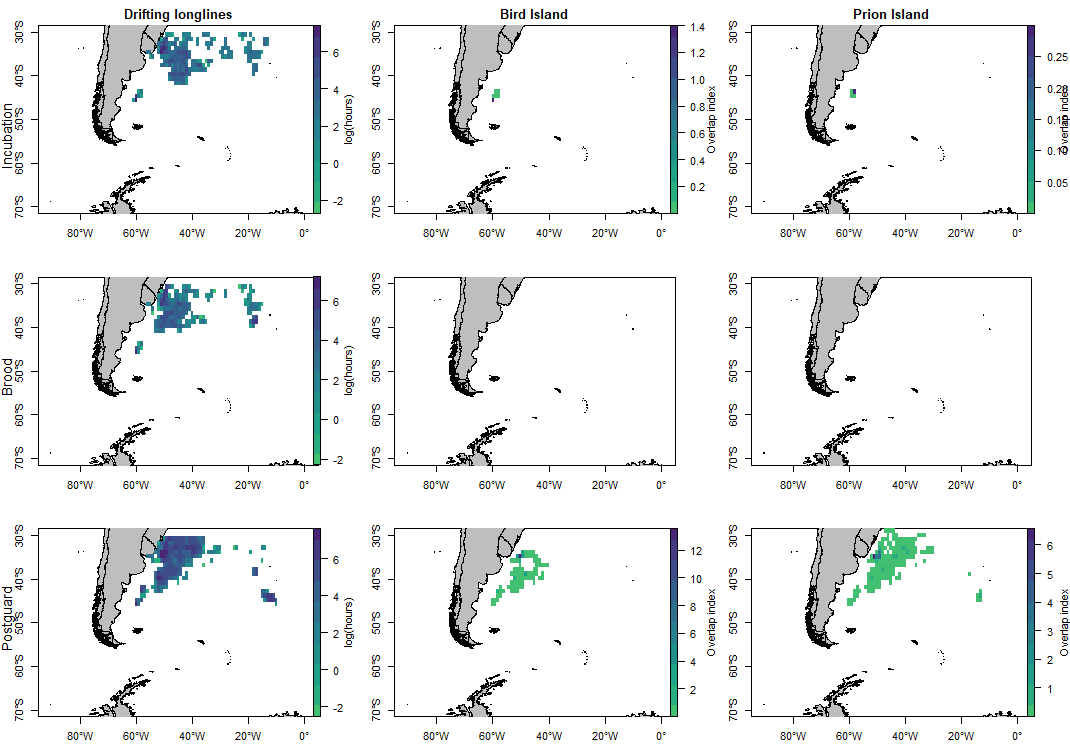


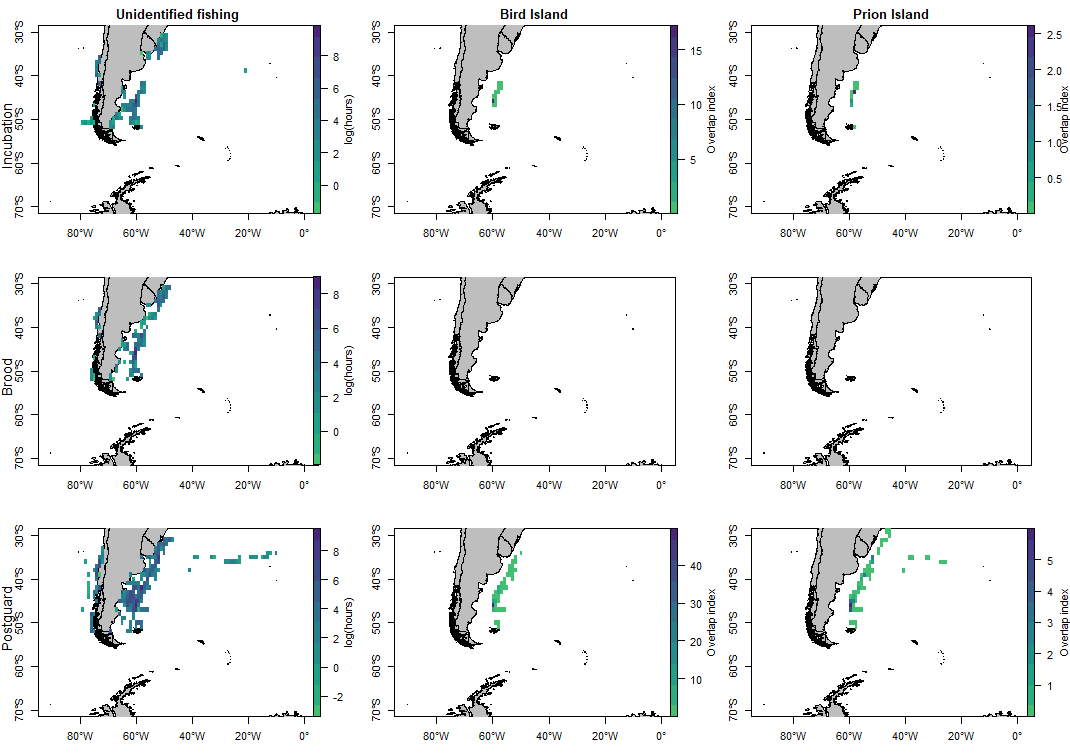


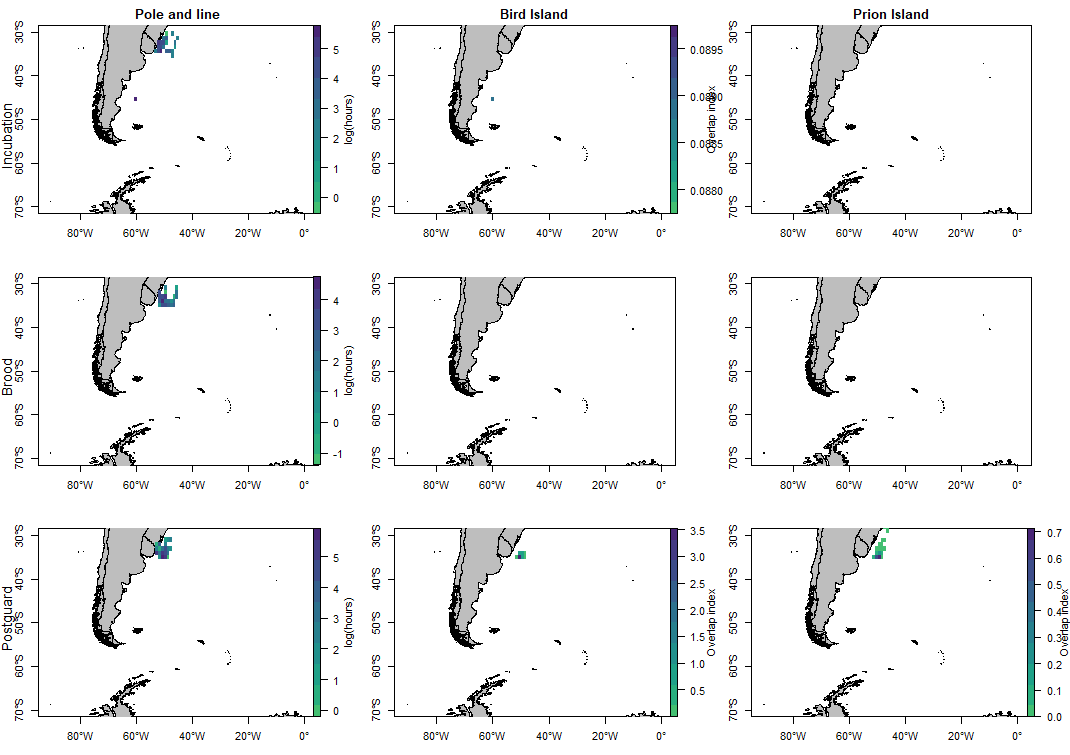


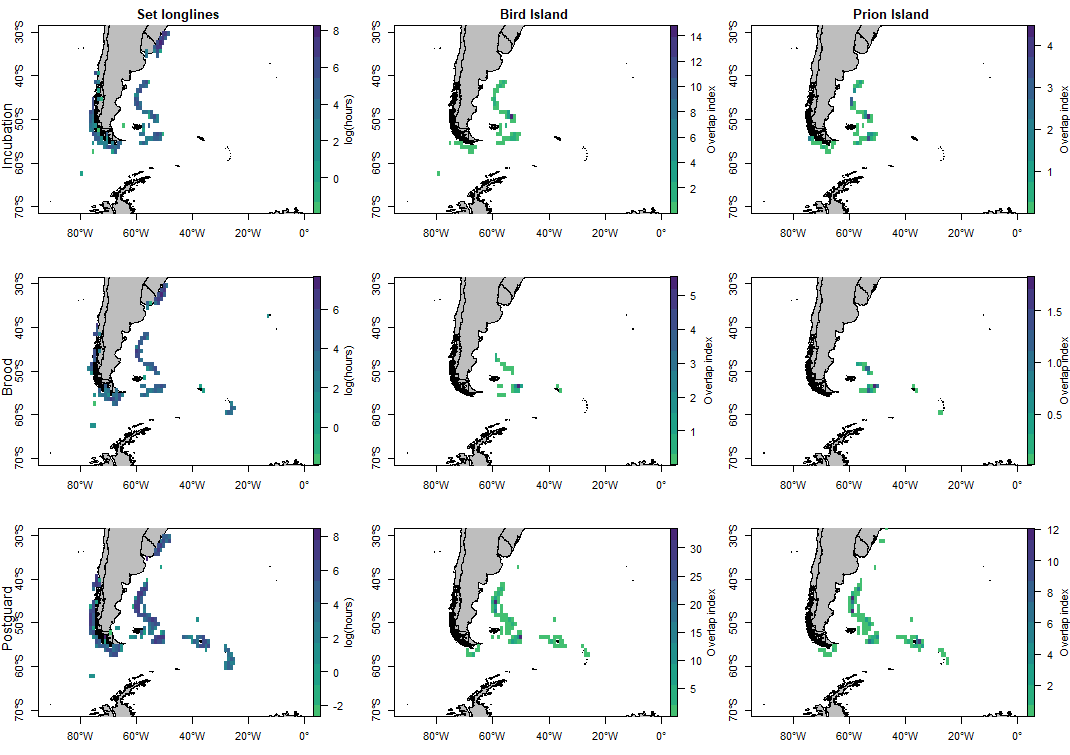


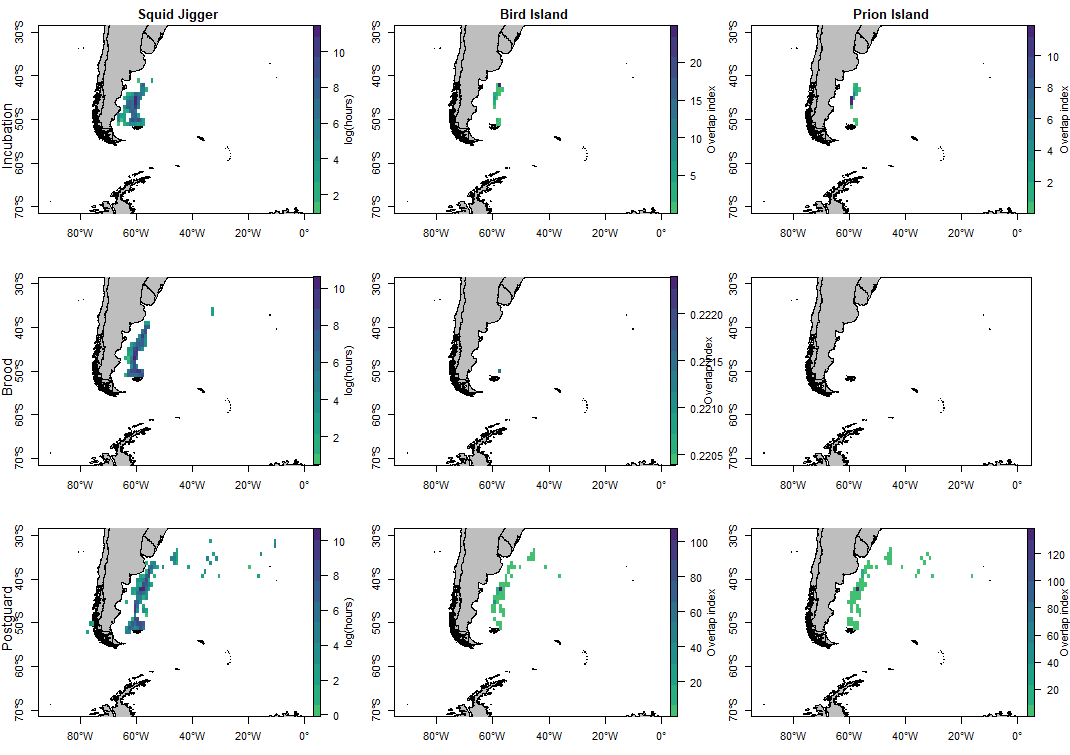


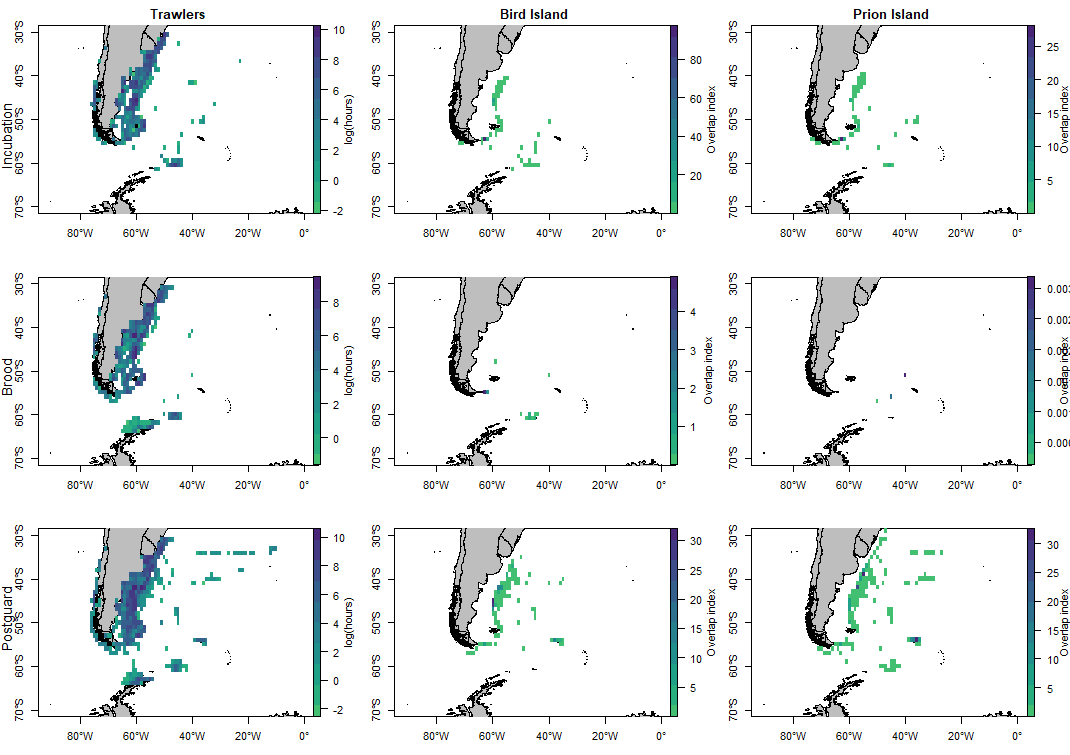


Figure S15. Spatial distribution of fishing effort by gear type recorded during incubation (9^th^ January - 29^th^ March), brood-guard (2^nd^ March - 18^th^ April) and post-guard (5^th^ April-18^th^ August) and areas of overlap with wandering albatrosses breeding at Bird Island and Prion Island. Fishing effort is described as the log of the total number of fishing hours during each stage of the breeding season.

S16. **Potential Mechanisms for the observed spatial segregation between colonies.**

*4.1 Competition*

Spatial segregation in the foraging distribution of birds from different colonies may be a response to density-dependent competition in central-place foragers (Wakefield et al. 2013, Wakefield et al. 2017, Bolton et al. 2019). This is a function of colony size and foraging distance, and may occur when the potential for competition is high, for example when prey are scarce, or colonies are large or close together. In contrast, in areas where prey are super-abundant, or if colonies are small or far apart, density-dependent competition is less likely (Wakefield et al. 2013).

The density-dependent competition hypothesis suggests that high overlap among colonies may occur where birds aggregate in highly productive areas that are remote from all colonies (Young et al. 2009, Wakefield et al. 2011, Wakefield et al. 2013, Bolton et al. 2019). We found high overlap on the Patagonian shelf-break, near the limit of the foraging range of birds from both colonies, supporting the idea that areas of high productivity further from the colony enable higher overlap. However, overlap along the North Scotia Arc and the Burdwood bank was also high, suggesting that mechanisms other than density-dependent competition are involved. Indeed, density-dependent competition does not explain why only birds breeding on Prion Island travelled east, and only birds breeding at Bird Island travelled to the Antarctic Peninsula and further west to feed. In some regions, spatial segregation is thought to occur because birds from small colonies avoid foraging in areas close to nearby larger colonies (Ainley et al. 2004, Cherel et al. 2017). However, in our study it was birds from Bird Island which did not travel past Prion Island, which is a much smaller colony (<30 pairs vs. 656 pairs at Bird Island in the 2021/22 season). There are a further ~140 breeding pairs on Albatross Island, close to Prion Island, that represent additional competition, but that still seems insufficient to explain the segregation. Given population trends are similar at Albatross and Prion islands (Rackete et al. 2021), we suspect that wandering albatrosses from the latter also travel east; however, this would need to be confirmed by further tracking. Although spatial segregation has been observed in species with small and large foraging ranges (Bolton et al. 2019), some wide-ranging pelagic seabirds nesting in neighbouring colonies do not show spatial segregation (e.g. Waggitt et al. 2014). As such, competition is not the only or necessarily the main driver of distributions.

*4.2 Influence of wind*

Wind affects the flight and foraging behaviour of many pelagic seabirds, which maximise efficiency by taking advantage of wind conditions (Spear and Ainley 1997, Weimerskirch et al. 2000, Ventura et al. 2020). Like many species, wandering albatrosses are more likely to travel with crosswinds than with head or tail winds, and are known to adjust their course in response to changing wind conditions (Weimerskirch et al. 2000, Wakefield et al. 2009, Richardson et al. 2018, Thorne et al. 2023). Wind strength and direction can also influence the bearing of departure of birds from the colony (Grémillet et al. 2004, Clay et al. 2023). As such, one explanation for the spatial segregation that we observed at South Georgia is that wind conditions at the time of departure vary between colonies and influence the direction of travel of subsequent foraging trips. Prevailing wind conditions have been shown to drive spatial segregation in cape gannets *Morus capensis*, facilitating travel away from neighbouring colonies (Grémillet et al. 2004), but to our knowledge that is the only study linking spatial segregation with wind conditions in seabirds. In contrast, wandering albatrosses from Prion Island and Bird Island experienced predominantly south-westerly winds of similar strength at the time of departure, regardless of the breeding stage. The bearing of foraging trips was not dependent on the strength or direction of the wind at departure, except at Bird Island during post-guard when bearing was associated with wind speed for birds from both breeding sites, and wind direction for those from Bird Island. Consequently, given that spatial segregation was observed in all breeding stages whereas wind conditions were predominantly the same except during post-guard it is unlikely that the observed spatial segregation is a result of variation in localised wind conditions at the colony.

Although there is a clear mechanism by which wind could drive spatial segregation between colonies, the lack of supporting evidence in this study or others suggests that this occurs infrequently. In contrast, differential responses to wind conditions as a result of sexual size dimorphism have been shown frequently to drive spatial segregation between male and female albatrosses (Phillips et al. 2004, Pereira et al. 2018, Clay et al. 2020). Female wandering albatrosses weigh less and have a lower wing loading than males, potentially leading to habitat partitioning. Consequently, females are more likely to exploit subtropical and tropical waters where winds are lighter, and males more likely to exploit more southerly waters where winds are stronger (Xavier and Croxall 2005, Froy et al. 2015). The sex of individuals at Prion Island was unknown and so we cannot test whether sexual size dimorphism influenced their distributions. Even if that were the case, it is important to note that the spatial segregation that we observed among colonies was primarily longitudinal rather than latitudinal, and so does not fit the usual pattern associated with sexual size dimorphism. Nor does it seem likely that the spatial segregation reflected a bias towards particular age classes at one of the colonies. There are weak effects of age on distribution of wandering albatrosses at South Georgia (Xavier et al. 2004, Froy et al. 2015, Clay et al. 2018), and at Prion Island, we only tracked 10 individuals. However, many individuals were tracked from Bird Island during each phase of the breeding period, including good samples of both sexes and of young, mid-aged and old birds. Moreover, our results are consistent with previous tracking of wandering albatrosses at Bird Island in multiple years which indicate that trips to the east of South Georgia are very uncommon (Xavier et al. 2004, Froy et al. 2015, Pereira et al. 2018).

*4.3 Habitat specialisation*

Habitat specialisation has the potential to drive spatial segregation if the preference for different habitat characteristics leads to foraging in different locations. Habitat preferences of seabirds frequently differ between colonies, age or sex classes (Wakefield et al. 2011, Torres et al. 2015, Clay et al. 2016), though it can be challenging to distinguish whether this variability is proximate or ultimate in most cases. Our study provides a valuable opportunity to investigate whether spatial segregation between neighbouring colonies may be driven by habitat specialisation.

Our habitat models performed well and identified very similar habitat preferences for birds breeding at Prion Island and Bird Island. Birds from both populations preferred shallower water close to breeding colonies with sea-surface temperatures of 0-20°C during all phases of the breeding season. During incubation, birds from Bird Island also favoured habitats with higher eddy kinetic energy, whereas those from Prion Island preferred either low or high eke. All of these covariates are frequently included in habitat models for pelagic seabirds and are indicative of areas of high productivity (Wakefield et al. 2011, Scales et al. 2016, Warwick-Evans et al. 2021). Our models also indicate that in areas where segregation was observed both east and west of South Georgia, the habitat characteristics are suitable for birds from both colonies.

The southwest Atlantic is one of the most productive regions of the Southern Ocean and includes extensive areas of high primary productivity and predictable prey availability within the foraging ranges of wandering albatrosses from South Georgia (Croxall and Wood 2002, Whitehouse et al. 2012, Matano et al. 2019). During incubation and post-guard, when foraging ranges are most extensive, our models predicted high occurrence at the Patagonian shelf-break, the North Scotia Arc, the Mid-Atlantic Ridge and the southern Argentine Basin, as well as the Subtropical Front during post-guard only. However, during brood-guard when foraging ranges are more constrained, areas of high predicted occurrence were limited to features nearer the archipelago such as the North Scotia Arc. Areas of high occurrence coincide with areas where primary productivity and prey availability are likely to be sufficiently high and predictable to counteract the costs of travel. Our models also indicate suitable foraging habitats in other areas of high productivity, such as the Antarctic Peninsula, and the South America-Antarctic Ridge. It is not clear why these were visited by individuals from just one colony. Prey species can differ between habitats (Xavier et al. 2004, Pereira et al. 2018), but it seems unlikely that prey preferences *per se* of wandering albatrosses would differ among colonies, although diet data only exists for birds breeding at Bird Island. Overall, given that habitat preferences for birds from both colonies are very similar, there is little indication that habitat specialisation drives spatial segregation within this island group.

*4.4 Information sharing, memory and cultural evolution*

In the absence of a convincing alternative involving competition, habitat specialisation or local wind patterns at the colony, another explanation for spatial segregation is social learning and the past experience of each individual, given the suggestion in other studies that information exchanged at the colony can result in the cultural evolution of foraging patterns (Wakefield et al. 2013, Morinay et al. 2023). Indeed, one of the benefits of colonial breeding is the transfer of information between individuals, which may facilitate group foraging (Grémillet et al. 2004, Weimerskirch et al. 2010, Wakefield et al. 2013, Sutton et al. 2017). It is plausible that these two populations are descended from two founder populations with different at-sea distributions, though this would need to be confirmed by a comparison of gene flow and population genetic structure. An alternative is that memorised personal information alone leads to spatial segregation between colonies, independent of the transfer of social information (Aarts et al. 2021). However, that seems less likely in our study because many wandering albatrosses remain in the southwest Atlantic from fledging until they first return to the colony, when they feed over very large areas that potentially encompass most or all of those used by breeding adults from both Bird Island and Prion Island. As such, it is not clear why birds from Bird Island would not continue to use the suitable habitat east of South Georgia even after recruitment. Instead, we suggest that cultural foraging patterns may have emerged that differ among the two populations.

Young, naïve birds are known to follow more experienced adults in other species (Ward and Zahavi 1973, Votier et al. 2011, Bolton et al. 2019). The recruitment process takes a few years, with individual wandering albatrosses arriving earlier and spending increasing time at the colony until they breed for the first time (Pickering 1989). It is therefore possible that in those formative years, when immatures are constrained to forage from a central place, that they develop a more restricted range, potentially following established breeders to areas specific to that colony. Although this remains speculative in the absence of further study, it is perhaps the most plausible explanation for segregation of foraging areas that we observed.

Aarts, G., E. Mul, J. Fieberg, S. Brasseur, J. A. van Gils, J. Matthiopoulos, and L. Riotte-Lambert. 2021. Individual-level memory is sufficient to create spatial segregation among neighboring colonies of central place foragers. The American Naturalist **198**:E37-E52.

Ainley, D. G., C. A. Ribic, G. Ballard, S. Heath, I. Gaffney, B. J. Karl, K. J. Barton, P. R. Wilson, and S. Webb. 2004. Geographic structure of Adélie Penguin populations: Overlap in colony‐specific foraging areas. Ecological Monographs **74**:159-178.

Bolton, M., G. Conolly, M. Carroll, E. D. Wakefield, and R. Caldow. 2019. A review of the occurrence of inter‐colony segregation of seabird foraging areas and the implications for marine environmental impact assessment. Ibis **161**:241-259.

Cherel, Y., J. C. Xavier, S. De Grissac, C. Trouvé, and H. Weimerskirch. 2017. Feeding ecology, isotopic niche, and ingestion of fishery-related items of the wandering albatross *Diomedea exulans* at Kerguelen and Crozet Islands. Marine Ecology Progress Series **565**:197-215.

Clay, T. A., P. Hodum, E. Hagen, and M. d. L. Brooke. 2023. Adjustment of foraging trips and flight behaviour to own and partner mass and wind conditions by a far-ranging seabird. Animal Behaviour **198**:165-179.

Clay, T. A., R. Joo, H. Weimerskirch, R. A. Phillips, O. Den Ouden, M. Basille, S. Clusella‐Trullas, J. D. Assink, and S. C. Patrick. 2020. Sex‐specific effects of wind on the flight decisions of a sexually dimorphic soaring bird. Journal of Animal Ecology **89**:1811-1823.

Clay, T. A., A. Manica, P. G. Ryan, J. R. Silk, J. P. Croxall, L. Ireland, and R. A. Phillips. 2016. Proximate drivers of spatial segregation in non-breeding albatrosses. Scientific reports **6**:29932.

Clay, T. A., E. J. Pearmain, R. A. McGill, A. Manica, and R. A. Phillips. 2018. Age‐related variation in non‐breeding foraging behaviour and carry‐over effects on fitness in an extremely long‐lived bird. Functional Ecology **32**:1832-1846.

Croxall, J., and A. Wood. 2002. The importance of the Patagonian Shelf for top predator species breeding at South Georgia. Aquatic Conservation: Marine Freshwater Ecosystems **12**:101-118.

Dickens, W., A. Graham, J. Smith, J. Dowdeswell, R. D. Larter, C.-D. Hillenbrand, P. N. Trathan, J. E. Arndt, and G. Kuhn. 2014. A new bathymetric compilation for the South Orkney Islands region, Antarctic Peninsula (49 degrees-39 degrees W to 64 degrees-59 degrees S): Insights into the glacial development of the continental shelf. GEOCHEMISTRY GEOPHYSICS GEOSYSTEMS **15**:2494-2514.

Froy, H., S. Lewis, P. Catry, C. M. Bishop, I. P. Forster, A. Fukuda, H. Higuchi, B. Phalan, J. C. Xavier, and D. H. Nussey. 2015. Age-related variation in foraging behaviour in the wandering albatross at South Georgia: no evidence for senescence. PLoS One **10**:e0116415.

Grémillet, D., G. Dell¹Omo, P. G. Ryan, G. Peters, Y. Ropert-Coudert, and S. J. Weeks. 2004. Offshore diplomacy, or how seabirds mitigate intra-specific competition: a case study based on GPS tracking of Cape gannets from neighbouring colonies. Marine Ecology Progress Series **268**:265-279.

Matano, R. P., E. D. Palma, and V. Combes. 2019. The Burdwood bank circulation. Journal of Geophysical Research: Oceans **124**:6904-6926.

Morinay, J., L. Riotte‐Lambert, G. Aarts, F. De Pascalis, S. Imperio, M. Morganti, C. Catoni, G. Assandri, S. Ramellini, and D. Rubolini. 2023. Within‐colony segregation of foraging areas: from patterns to processes. Oikos **2023**:e09926.

Pereira, J. M., V. H. Paiva, R. A. Phillips, and J. C. Xavier. 2018. The devil is in the detail: small-scale sexual segregation despite large-scale spatial overlap in the wandering albatross. Marine Biology **165**:1-16.

Phillips, R., J. Silk, B. Phalan, P. Catry, and J. Croxall. 2004. Seasonal sexual segregation in two Thalassarche albatross species: competitive exclusion, reproductive role specialization or foraging niche divergence? Proceedings of the Royal Society of London. Series B: Biological Sciences **271**:1283-1291.

Pickering, S. 1989. Attendance patterns and behaviour in relation to experience and pair‐bond formation in the Wandering Albatross *Diomedea exulans* at South Georgia. Ibis **131**:183-195.

Richardson, P. L., E. D. Wakefield, and R. A. Phillips. 2018. Flight speed and performance of the wandering albatross with respect to wind. Movement ecology **6**:1-15.

Scales, K. L., P. I. Miller, S. N. Ingram, E. L. Hazen, S. J. Bograd, and R. A. Phillips. 2016. Identifying predictable foraging habitats for a wide‐ranging marine predator using ensemble ecological niche models. Diversity and Distributions **22**:212-224.

Spear, L. B., and D. G. Ainley. 1997. Flight behaviour of seabirds in relation to wind direction and wing morphology. Ibis **139**:221-233.

Sutton, G. J., A. J. Hoskins, M. Berlincourt, and J. P. Arnould. 2017. Departure time influences foraging associations in little penguins. PLoS One **12**:e0182734.

Thorne, L., T. Clay, R. Phillips, L. Silvers, and E. Wakefield. 2023. Effects of wind on the movement, behavior, energetics, and life history of seabirds. Marine Ecology Progress Series **723**:73-117.

Torres, L. G., P. J. Sutton, D. R. Thompson, K. Delord, H. Weimerskirch, P. M. Sagar, E. Sommer, B. J. Dilley, P. G. Ryan, and R. A. Phillips. 2015. Poor transferability of species distribution models for a pelagic predator, the grey petrel, indicates contrasting habitat preferences across ocean basins. PLoS One **10**:e0120014.

Ventura, F., J. P. Granadeiro, O. Padget, and P. Catry. 2020. Gadfly petrels use knowledge of the windscape, not memorized foraging patches, to optimize foraging trips on ocean-wide scales. Proceedings of the Royal Society B **287**:20191775.

Votier, S. C., W. J. Grecian, S. Patrick, and J. Newton. 2011. Inter-colony movements, at-sea behaviour and foraging in an immature seabird: results from GPS-PPT tracking, radio-tracking and stable isotope analysis. Marine Biology **158**:355-362.

Waggitt, J., M. Briffa, W. J. Grecian, J. Newton, S. C. Patrick, C. Stauss, and S. C. Votier. 2014. Testing for sub-colony variation in seabird foraging behaviour: ecological and methodological consequences for understanding colonial living. Marine Ecology Progress Series **498**:275-285.

Wakefield, E. D., T. W. Bodey, S. Bearhop, J. Blackburn, K. Colhoun, R. Davies, R. G. Dwyer, J. A. Green, D. Grémillet, and A. L. Jackson. 2013. Space partitioning without territoriality in gannets. Science **341**:68-70.

Wakefield, E. D., E. Owen, J. Baer, M. J. Carroll, F. Daunt, S. G. Dodd, J. A. Green, T. Guilford, R. A. Mavor, and P. I. Miller. 2017. Breeding density, fine‐scale tracking and large‐scale modeling reveal the regional distribution of four seabird species. Ecological Applications.

Wakefield, E. D., R. A. Phillips, J. Matthiopoulos, A. Fukuda, H. Higuchi, G. J. Marshall, and P. N. Trathan. 2009. Wind field and sex constrain the flight speeds of central‐place foraging albatrosses. Ecological Monographs **79**:663-679.

Wakefield, E. D., R. A. Phillips, P. N. Trathan, J. Arata, R. Gales, N. Huin, G. Robertson, S. M. Waugh, H. Weimerskirch, and J. Matthiopoulos. 2011. Habitat preference, accessibility, and competition limit the global distribution of breeding Black‐browed Albatrosses. Ecological Monographs **81**:141-167.

Ward, P., and A. Zahavi. 1973. The importance of certain assemblages of birds as “information‐centres” for food‐finding. Ibis **115**:517-534.

Warwick-Evans, V., J. A Santora, J. J. Waggitt, P. N. Trathan, and S. Votier. 2021. Multi-scale assessment of distribution and density of procellariiform seabirds within the Northern Antarctic Peninsula marine ecosystem. ICES Journal of Marine Science **78**:1324-1339.

Weimerskirch, H., S. Bertrand, J. Silva, J. C. Marques, and E. Goya. 2010. Use of social information in seabirds: compass rafts indicate the heading of food patches. PLoS One **5**:e9928.

Weimerskirch, H., T. Guionnet, J. Martin, S. A. Shaffer, and D. Costa. 2000. Fast and fuel efficient? Optimal use of wind by flying albatrosses. Proceedings of the Royal Society of London. Series B: Biological Sciences **267**:1869-1874.

Whitehouse, M., A. Atkinson, R. Korb, H. Venables, D. Pond, and M. Gordon. 2012. Substantial primary production in the land-remote region of the central and northern Scotia Sea. Deep Sea Research Part II: Topical Studies in Oceanography **59**:47-56.

Xavier, J., P. Trathan, J. Croxall, A. Wood, G. Podesta, and P. Rodhouse. 2004. Foraging ecology and interactions with fisheries of wandering albatrosses (*Diomedea exulans*) breeding at South Georgia. Fisheries Oceanography **13**:324-344.

Xavier, J. C., and J. P. Croxall. 2005. Sexual differences in foraging behaviour and diets: a case study of wandering albatrosses. Sexual segregation in vertebrates:74-91.

Young, L. C., C. Vanderlip, D. C. Duffy, V. Afanasyev, and S. A. Shaffer. 2009. Bringing home the trash: do colony-based differences in foraging distribution lead to increased plastic ingestion in Laysan albatrosses? PLoS One **4**:e7623.
